# Systematic Selection of High‐Affinity ssDNA Sequences to Carbon Nanotubes

**DOI:** 10.1002/advs.202308915

**Published:** 2024-06-25

**Authors:** Dakyeon Lee, Jaekang Lee, Woojin Kim, Yeongjoo Suh, Jiwoo Park, Sungjee Kim, YongJoo Kim, Sunyoung Kwon, Sanghwa Jeong

**Affiliations:** ^1^ School of Biomedical Convergence Engineering Pusan National University Yangsan 50612 Republic of Korea; ^2^ Department of Chemistry Pohang University of Science and Technology Pohang 37673 Republic of Korea; ^3^ Department of Materials Science and Engineering Kookmin University Seoul 02707 Republic of Korea; ^4^ Department of Materials Science and Engineering Korea University Seoul 02841 Republic of Korea; ^5^ Center for Artificial Intelligence Research Pusan National University Busan 46241 Republic of Korea

**Keywords:** binding affinity, machine learning, molecular dynamics, selection, single‐walled carbon nanotube, ssDNA

## Abstract

Single‐walled carbon nanotubes (SWCNTs) have gained significant interest for their potential in biomedicine and nanoelectronics. The functionalization of SWCNTs with single‐stranded DNA (ssDNA) enables the precise control of SWCNT alignment and the development of optical and electronic biosensors. This study addresses the current gaps in the field by employing high‐throughput systematic selection, enriching high‐affinity ssDNA sequences from a vast random library. Specific base compositions and patterns are identified that govern the binding affinity between ssDNA and SWCNTs. Molecular dynamics simulations validate the stability of ssDNA conformations on SWCNTs and reveal the pivotal role of hydrogen bonds in this interaction. Additionally, it is demonstrated that machine learning could accurately distinguish high‐affinity ssDNA sequences, providing an accessible model on a dedicated webpage (http://service.k‐medai.com/ssdna4cnt). These findings open new avenues for high‐affinity ssDNA‐SWCNT constructs for stable and sensitive molecular detection across diverse scientific disciplines.

## Graphical abstract

To elucidate the relationship between DNA bases and single‐walled carbon nanotubes (SWCNTs), a high‐throughput approach is used to enrich DNA sequences with high affinity for SWCNT surfaces from a large random DNA library. Molecular dynamics (MD) simulations based on DNA affinity quantification improve the understanding of this interaction and enable binding affinity predictions through machine learning.

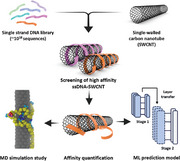

## 1 Introduction

Single‐walled carbon nanotubes (SWCNTs) have generated tremendous interest in various fields, including nanoelectronics^[^ ^1^ ^]^ and biotechnology^[^ ^2^ ^]^ due to their unique physical and chemical properties. SWCNTs are graphene sheets rolled into a cylindrical shape, creating a nanoscale tube with a high aspect ratio. SWCNT chirality originates from orientation as they are rolled up in a graphene sheet via a chiral vector (n,m).^[^ ^3^ ^]^ The semiconductor SWCNTs are determined by (n,m), where larger values of (n,m) correspond to larger diameters and the emission of longer near‐infrared wavelengths. They possess excellent mechanical, electrical, and optical properties, making them highly attractive for various applications. However, their insolubility and potential toxicity have limited their practical use in biomedical and nanoelectronics applications. To overcome these challenges, researchers have been exploring various strategies to functionalize and modify SWCNT surfaces.^[^ ^4^ ^]^

One promising approach is the use of single‐stranded DNA (ssDNA) as a wrapping surfactant for SWCNTs.^[^ ^5^ ^]^ ssDNA contains both a hydrophilic region and hydrophobic nitrogenous bases: adenine (A), cytosine (C), guanine (G), and thymine (T). ssDNA‐wrapped SWCNTs have shown great potential in biomedical applications, including drug delivery, gene therapy, and biosensors.^[^ ^6^ ^]^ Through precise hybridization control, millions of semiconducting ssDNA‐SWCNT constructs can be densely and uniformly assembled at even pitches for nanoelectronics.^[^ ^1d^ ^]^ ssDNA‐SWCNT constructs contain ssDNA molecules non‐covalently attached to the SWCNT surface through π‐π stacking, with base‐dependent binding strength.^[^ ^3^, ^7^ ^]^ The base composition and sequence can influence the strength and specificity of ssDNA wrapping as well as the conformation and packing density of the ssDNA molecules on SWCNTs.^[^ ^8^ ^]^ For example, Tu et al. suggested that specific ssDNA sequences can create 3D folding structures on the SWCNT surface, and these “recognition sequences” can be utilized for SWCNT chirality‐dependent sorting via chromatographic purification.^[^ ^3^ ^]^ Van der Waals interactions and intermolecular hydrogen bonds contribute to the formation of stable folding structures. Albertorio et al. experimentally quantified the nucleobase binding strength with SWCNTs in aqueous solutions by measuring the dissociated DNA fraction at high temperatures and revealed the order G>C>A>T.^[^ ^8a^ ^]^ Another experiment performed by peeling off multiple ssDNA molecules via single‐molecule force spectroscopy reported a different trend of nucleobase binding interactions in the order T>A>G>C.^[^ ^8b^ ^]^ The interaction between ssDNA and SWCNT significantly depends on the DNA sequence; it has been shown that a variance of only one base induced a 20‐fold enhancement in the binding strength.^[^ ^8c^ ^]^

Molecular dynamics (MD) simulation studies have been conducted to gain insight into the DNA‐wrapped SWCNT structures.^[^ ^7^, ^8^, ^9^ ^]^ Roxbury et al. simulated the right‐handed helical wrapping of 12‐nt ssDNA molecules on SWCNTs and quantitatively measured the driving force of helical wrapping based on hydrogen bonding, conformation entropy, electrostatic repulsion, and DNA bending.^[^ ^8c^ ^]^ Tu et al. also showed that the recognition sequence formed a stable structure on the SWCNT through hydrogen bonding, like the β‐sheet motif in proteins.^[^ ^3^ ^]^ Alizadehmojarad et al. proposed that the DNA sequence and preferential conformational (e.g., ring and helix) changes on the SWNCTs correlated with (*n*, *n*+2) nucleotides from intramolecular hydrogen bonds, influencing the binding affinity of short 12‐nt ssDNA molecules.^[^ ^9^ ^]^

In all‐atom simulations, Johnson et al. found that, in an aqueous solution, DNA‐CNT interaction is mediated by attractive π–π stacking, causing ssDNA to stack onto the SWCNT sidewall.^[^ ^10^ ^]^ They investigated the stability of poly GT oligonucleotides proposed by Zheng et al.^[^ ^11^ ^]^ It was observed that these oligonucleotides prefer to stack onto the SWCNT surface individually rather than forming dimers.^[^ ^10^ ^]^ Additionally, they discovered that the (GT)_7_‐SWCNT hybrid conformation is a global minimum with a nonhelical loop structure from the free energy landscape.^[^ ^12^ ^]^ Furthermore, they demonstrated that base–CNT binding is dominated by π–π stacking interactions with solvent and entropic effects playing a minor role.^[^ ^13^ ^]^

Recent studies have employed machine learning (ML) techniques to tackle two key aspects of DNA‐SWCNT interactions: 1) chiral sorting of SWCNTs using ssDNA strands,^[^ ^14^ ^]^ and 2) elucidating the molecular recognition mechanisms within the ssDNA‐SWCNT corona phase.^[^ ^15^ ^]^ Lin et al. identified DNA sequences for sorting the SWCNT of single‐chirality, initiating ML training with roughly 100 sequences of short 5‐nt with the combination of G/C.^[^ ^14b^ ^]^ Gong et al. demonstrated ML‐based molecular recognition prediction to identify the ssDNA sequence‐dependent 3D complexity in ssDNA‐SWCNT constructs.^[^ ^15^ ^]^

ML models have also been used to understand and improve the synthesis of different nanomaterials functionalized with various DNA sequences.^[^ ^16^ ^]^ For example, Copp et al. successfully classified the specific base motifs of DNA to fabricate the desired fluorescent wavelength and brightness of silver nanoclusters through SVM and random forest.^[^ ^16a^ ^]^

A deeper understanding of the structures and properties of SWCNT coatings is essential for their potential biological applications in biosensors and drug delivery. However, previous studies focused on relatively short ssDNAs (shorter than 12‐nt) and simple sequences such as DNAs with repeated motifs (e.g., (GT)_6_ and (TAT)_4_).^[^ ^6^, ^8^, ^17^ ^]^ In this work, we systematically selected high‐affinity 30‐nt ssDNA sequences from a random DNA library of up to 4^30^ (≈10^18^) sequences. Selected DNA sequences are rich in A and C bases and lack T and G bases. In this study, we verified the successful selection of high‐affinity ssDNA sequences after six rounds of iteration by monitoring the kinetics of competitive surfactant replacement from ssDNA to sodium cholate (SC). MD simulations of high‐ and low‐affinity ssDNAs demonstrate the contribution of hydrogen bonding and sequence patterns upon binding affinity. Furthermore, we employed several machine‐learning models to interpret the sequence patterns for binding affinity selection and created a freely accessible online service that can predict the binding affinity of ssDNA sequences to SWCNT. Through a combination of experimental, computational, and machine learning approaches, we have gained valuable insights into the sequence‐dependent binding affinity and stability of ssDNA‐SWCNT constructs.

## 2 Results

### 2.1 Selection of ssDNAs with High Binding Affinity to SWCNTs

**Figure 1:**
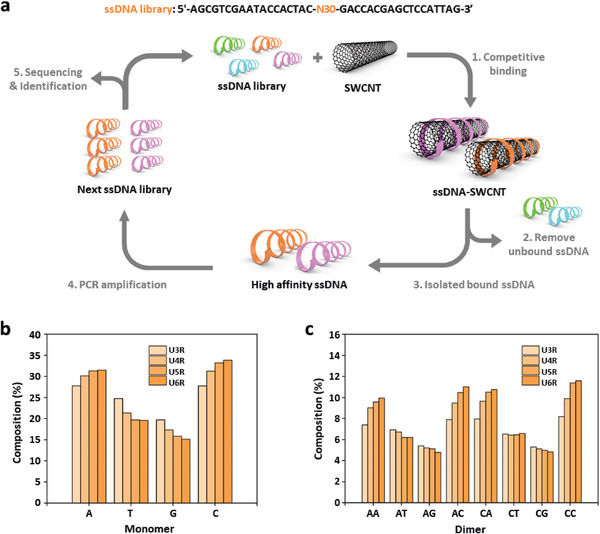
Selection of high‐affinity ssDNA sequences on SWCNT surfaces. a) Schematic representation of the selection process for high‐affinity ssDNA sequences on SWCNT surfaces. Population of b) ATGC monomer, and c) dimer motifs which contain the A and C bases in the top 20,000 sequences from the DNA library produced in rounds 3, 4, 5, and 6.

The initial random 66‐nt ssDNA library (Round 0) consists of random 30‐nt where 4^30^ (≈1.1×10^18^) unique sequences could be produced, flanked by two 18‐nt primer regions for polymerase chain reaction (PCR) amplification (**Figure** **1**a). Next, 10 µg HiPCo SWCNTs were tip‐sonicated in PBS buffer solution with excessive 1 mg of the ssDNA library. High‐affinity ssDNA sequences could be adsorbed onto the SWCNT surface to generate colloidal ssDNA‐wrapped SWCNT constructs. The unbound ssDNA molecules were removed via centrifugal filtration. Surface‐bound ssDNAs were then isolated and amplified by PCR to prepare the next‐round ssDNA library (Round 1). We repeated this process up to round 6 to select a high‐affinity ssDNA library. The DNA libraries at rounds 3, 4, 5, and 6 were subjected to high‐throughput sequencing using the Illumina Novaseq 6000 platform, as previously described.^[^ ^18^ ^]^

At first, we investigated the k‐mer base identity preference in the 20,000 most numerous sequences at each round. Following six rounds of selection, the monomer trend showed enrichment of C and A and a decline in the content of G and T (Figure 1b). In the sixth round, we observed a monomer composition of 33.5% C, 31.6% A, 19.6% G, and 15.3% T. The top 100 sequences, sorted by frequency of unique sequences in each library, also showed the same trend, with similar ATGC compositions (Figure S1, Supporting Information). Dimer preference in the top 200000 numerous sequences presented similar trends; the content of AA, AC, CA, and CC increased while that of AG, AT, and CG slightly decreased (Figure 1c; Figure S2, Supporting Information). These results suggest that the high‐affinity selection process induced the enrichment of A and C and the decrease in the content of G and T.

### 2.2 Quantitative Characterization of the Sequence‐Dependent ssDNA Binding Affinity to SWCNT

**Figure 2:**
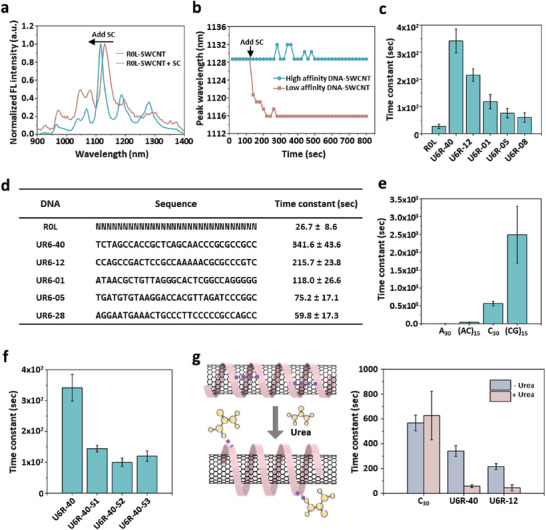
Characterization of sequence‐dependent ssDNA binding affinity onto SWCNTs. a) Fluorescence (FL) spectra of R0L‐SWCNTs before and after incubation with 0.02 wt% sodium cholate (SC). b) Time‐dependent spectral shift of the FL peak around 1129 nm of R0L DNA‐SWCNTs (low affinity) and U6R‐40 DNA‐SWCNTs (high affinity) with 0.02 wt% SC. c) Time constants of the spectral shift for high‐affinity ssDNA molecules (U6R‐01, 05, 12, 28, and 40) and low‐affinity R0L with 0.05 wt% SC. The time constant for the displacement of ssDNA molecules from a surface by SC surfactants is a measure of the kinetic stability of ssDNA on the surface. d) The table of time constant values for R0L and top 5 sequences. e) Comparison of the binding affinity of A_30_, C_30_, (AC)_15_, (GC)_15_ ssDNA via 0.05 wt% SC displacement. f) Comparison of the binding affinity of U6R‐40 and three scrambled sequences (U6R‐40‐S1, S2, and S3) via 0.05 wt% SC displacement. g) Comparison of the binding affinity of C30, U6R‐40, and U6R‐12, via 0.05 wt% SC displacement with or without pre‐incubation with 3 M urea. Urea disrupts the intermolecular hydrogen bonds within ssDNA on the SWCNT surface.

To quantify the binding affinity of certain ssDNA sequences on SWCNTs, we performed surfactant displacement experiments on 47 sequences from our final library (round 6). The sequences from round 6 were chosen and denoted as U6R‐M (Mth place of round 6 in the descending order of frequency). The primer regions at each end were omitted to investigate the influence of the random 30‐nt regions only. Previous studies showed that high‐affinity surfactants like SC can displace ssDNA‐SWCNT constructs into SC‐SWCNT constructs.^[^ ^18^ ^]^ During surfactant replacement, the fluorescence peak wavelengths of the SWCNT displayed a time‐dependent blueshift (**Figure** **2**a). As a control example for the affinity of the initial random ssDNA molecule, fluorescence spectra of SWCNT constructs wrapped with 30‐nt from the initial ssDNA library (round 0; R0L‐SWCNT) showed a rapid blue‐shift in the presence of 0.02 wt% SC (Figure 2b). After adding SC, the fluorescence peak at 1129 nm, which mainly represents (9,4) chirality,^[^ ^9^, ^19^ ^]^ was completely blue‐shifted to 1115 nm for 3 min. Furthermore, SWCNT constructs wrapped with U6R‐40 showed no signs of spectral shift over 10 min, which indicates the high affinity of that sequence.

We leveraged the kinetics of surfactant displacement to quantify the binding affinity of each ssDNA sequence; a longer displacement time indicates a higher binding affinity. The change in the fluorescence peak representing (9,4) SWCNT chirality was observed to obtain the time constant of the surfactant displacement reaction, assuming first‐order exponential decay (Figure S3, Supporting Information). About fifty abundant sequences in the final (round 6) library were tested; 46 of the 47 sequences showed a higher affinity than R0L‐SWCNT constructs in 0.02 wt% SC (Table S1, Supporting Information). Because 18 of the 47 sequences tested did not show a significant spectral shift at 0.02 wt% SC, we did another experiment under a higher wt% of SC (0.05 wt%) to quantitively compare the binding affinity of each sequence (Table S2, Supporting Information). Unless otherwise specified, the time constant at 0.05 wt% SC conditions was utilized subsequently in this manuscript. The time displacement constants of the top 5 sequences with the highest affinity are shown in Figure 2c. U6R‐40 has the highest time constant of 341.6 ± 43.6 s, 12 times higher than that of the initial library (Figure 2c,d).

Our observations indicated a significant enhancement in affinity upon the incorporation of primer regions on both flanks of the DNA molecule. Under SC 0.05 wt% condition, the high‐affinity sequences U6R‐40, U6R‐12, U6R‐1, and U6R‐5 showed the increase of time constant in the presence of primer, but the hierarchy did not change (U6R‐40 > U6R‐12 > U6R‐1 > U6R‐5) (Table S3, Supporting Information). Low‐affinity sequences remain the significantly smaller time constant rather than high‐affinity ones despite the primer inclusion. This result suggests that the existence of the primer region does not alter the trends or utility of the identified central sequences.

We also investigated the effect of SWCNT chirality on the kinetics of surfactant displacement from DNA‐SWCNTs to SC‐SWCNTs. SWCNT can form a cylinder rolled up from a graphene sheet via a chiral vector (n,m), defining the “chirality” of a SWCNT.^[^ ^3^ ^]^ The structure of semiconductor (n,m)‐SWCNTs is determined by (n,m), where larger values of (n,m) correspond to larger diameters and the emission of longer near‐infrared wavelengths due to their smaller band structure. Each fluorescence peak indicates distinct SWCNT chirality in the photoluminescence (PL) spectrum (Figure S4, Supporting Information). During surfactant displacement, DNA‐SWCNT constructs have shown diameter‐dependent reaction kinetics. SWCNTs with large diameters showed faster fluorescence peak shifts than those with small diameters after treatment with 0.02 wt% SC. Moreover, replacing SC seems easier for SWCNTs with larger diameters than those with smaller diameters.

ssDNA wrapping on SWCNTs can generate sequence‐dependent 3D corona phases around SWCNTs.^[^ ^3^, ^20^ ^]^ These corona structures have contributed to the binding moiety for biomolecules and the chiral sorting of SWCNTs.^[^ ^3^ ^]^ In our investigation, we explored the kinetics of surfactant displacement along the SWCNT chirality from DNA‐SWCNTs to SC‐SWCNTs. Each fluorescence peak indicates distinct SWCNT chirality in the PL spectrum (Figure S4a, Supporting Information). During surfactant displacement, DNA‐SWCNT constructs exhibited diameter‐dependent reaction kinetics. SWCNTs with large diameters showed faster and larger fluorescence peak shifts than those with small diameters after treatment with 0.02 wt% SC (Figure S4b,c, Supporting Information). Considering the selection of specific nucleotide compositions (Figure 1b), we tested the displacement time constant for several sequences, including A_30_, (AC)_15_, C_30_, and (CG)_15_ (Figure 2e). The time constants of A_30_, (AC)_15_, and C_30_ increased as the C content increased. Surprisingly, the (CG)_15_ sequence showed the longest time constant, although it contained the same amount of C as the (AC)_15_ sequence.

We also hypothesized that the binding affinity of ssDNAs is not only correlated with the nucleotide composition but also with the nucleotide sequence. Three ssDNA‐SWCNT constructs were prepared from the sequence‐scrambled sequences of U6R‐40 (U6R‐40‐S1, U6R‐40‐S2, and U6R‐40‐S3). These constructs have the same A, T, G, and C composition, but the bases were arranged differently. U6R‐40‐S1, U6R‐40‐S2, and U6R‐40‐S3 showed much lower time constants than U6R‐40, at 144.3 ± 10.7, 99.7 ± 13.8, and 120.3 ± 17.2 s, respectively (Figure 2f). This result clearly shows the influence of the nucleotide sequence on their binding affinity to SWCNTs.

Intramolecular hydrogen bonding between nucleotide bases contributes to the binding strength of ssDNA on SWCNTs as DNA origami creates rigid 3D structures.^[^ ^21^ ^]^ After pre‐incubation in 3 M urea, a chaotropic agent that disrupts the hydrogen bonding network,^[^ ^22^ ^]^ the time constants of U6R‐12‐ and U6R‐40‐SWCNT constructs were reduced to 80% and 83% compared to the urea‐free experiments (Figure 2g). However, the time constant of C_30_‐SWCNT constructs exhibited little change. C_30_ does not have canonical complementary base pair sequences such as A‐T and G‐C pairs, but U6R‐12 and U6R‐40 do. ssDNA strand adhered onto the nanotube surface can be stabilized by Watson‐Crick (canonical) and non‐Watson‐Crick (non‐canonical) base pairing, along with π–π stacking interactions between DNA bases and the nanotube surface.^[^ ^7^ ^]^ The appropriate conformation of ssDNA might induce a number of interstitial base pairings that enhance the binding affinity. These results indicate that the binding affinity of ssDNA is strongly correlated with their nucleotide sequences and intramolecular hydrogen bonding in addition to the base composition.

### 2.3 Molecular Dynamics Simulation of ssDNA Adsorption to SWCNTs

**Figure 3:**
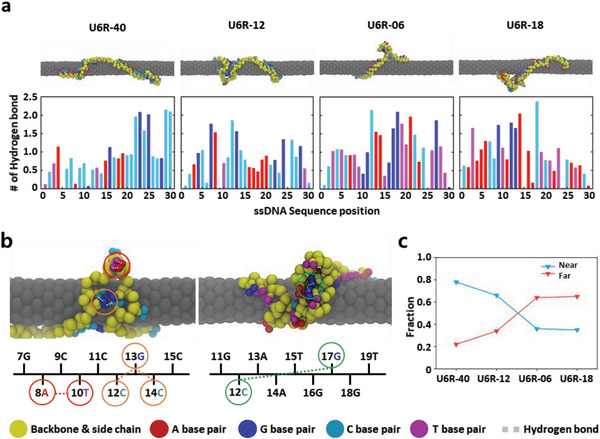
MD simulation of high‐affinity ssDNA adsorption on SWCNT surfaces. a) Snapshots of the U6R‐40, U6R‐12, U6R‐06, and U6R‐18 system after 500 ns of simulation. (Yellow beads represent the ssDNA backbone and the attached side chains. Cyan, blue, red, and magenta beads represent C, G, A, and T nucleobase, respectively). An average number of hydrogen bonds (H‐bond) formed during the last 200 ns between base pair beads of ssDNA and SWCNT are shown below. b) Magnified snapshots of U6R‐12 and U6R‐06 systems after 500 ns of simulation. Red circles represent the H‐bond between the 8A and 10T of U6R‐12; orange circles represent H‐bonds between 12C, 13G, and 14C of U6R‐12; and green circles represent H‐bonds between the 12C and 17G of U6R‐06. c) Percentages of “near H‐bonds” and “far H‐bonds” in the four ssDNA‐SWCNT systems. Near H‐bond % = Near H‐bond / (Near H‐bond + Far H‐bond). Far H‐bond % = Far H‐bond / (Near H‐bond + Far H‐bond).

We performed coarse‐grained molecular dynamics (CGMD) simulations of 30‐nt ssDNA oligomers adsorbing to SWCNTs using GROMACS with MARTINI force field 2.^[^ ^23^ ^]^ The MARTINI force field is widely recognized and utilized in molecular simulations due to its unique approach of representing groups of atoms as a single bead. We employed the 4‐to‐1, 3‐to‐1, and 2‐to‐1 mapping methods, which group four, three, and two heavy atoms into a single bead, respectively, to enable faster simulations compared to typical all‐atom force field‐based MD simulations.^[^ ^24^ ^]^ To investigate the sequence‐dependent interaction between ssDNA and SWCNTs, we employed armchair‐type SWCNTs with 1.2‐nm diameters and four ssDNA molecules in an aqueous environment: two high‐affinity (U6R‐12 and U6R‐40) and two low‐affinity ssDNA molecules (U6R‐06 and U6R‐18). After reaching equilibrium, each nucleobase of all four sequences showed negative van der Waals (vdW) energies to SWCNT (Figure S5, Supporting Information), demonstrating their positive binding affinity to the SWCNT surface. We also calculated the number of intramolecular hydrogen bonds among the ssDNA molecules at each base position and plotted the average hydrogen bond numbers during the last 200 ns of the simulation (**Figure** **3**a; Movies S1–S4, Supporting Information).

The negative vdW energies of U6R‐40, U6R‐12, U6R‐06, and U6R‐18 indicate their positive binding affinity to SWCNTs. The overall vdW energies showed a clear correlation with the time constants of the ssDNAs in each experiment (Figure S5, Supporting Information). Position‐specific vdW interactions tend to weaken when the base position prefers to make many hydrogen bonds. Hence, it can be inferred that when a base pair in the ssDNA forms intramolecular hydrogen bonding, it detaches from the SWCNT surface, adopting a secondary structure (Figure 3b). The formation of this secondary structure weakens the vdW interaction. Weakly bound secondary structures can be found in the bases of U6R‐12 (8A and 10T) and U6R‐06 (12C and 17G) (red circle and green circle in Figure 3b, respectively). However, in the case of U6R‐12 (12C, 13G, and 14C), the correlation between vdW energy and the number of hydrogen bonds is the opposite, with strong SWCNT binding through intramolecular hydrogen bonds near the SWCNT surface (orange circles in Figure 3b). These exceptional cases typically arise for simulating DNA sequences with consecutive C and G bases. Interestingly, in regions where C and G appear consecutively, even when these base pairs form intramolecular hydrogen bonds, they tend to attach to the SWCNT and are not detached from its surface.

Furthermore, we examined the distance between the SWCNT surface and the backbone bases that form intramolecular hydrogen bonding (Figure 3c). We classified these bases into two groups: “near H‐bond,” with distances below 6 Å, and “far H‐bond,” with distances above 6 Å. DNA bases with high affinity exhibit an increase in the proportion of “near H‐bonds” and a decrease in the proportion of “far H‐bonds”; sequences with low affinity showed the opposite trend.

The near H‐bonds within 6 Å from the SWCNT surface exhibit high adsorption energy and hydrogen bonding, stabilizing the structure through intramolecular interaction and forming a rigid configuration on the SWCNT. Conversely, far H‐bonds beyond 6 Å also contribute to a rigid tertiary structure via hydrogen bonding but exhibit weaker adsorption energy due to its formation away from the surface.

### 2.4 Prediction of High Binding ssDNA‐SWCNT via Machine Learning Model

Previous studies have used traditional machine learning models such as SVM, random forest, and logistic regression to predict the binding of DNA and SWCNT.^[^ ^14^, ^25^ ^]^ DNA sequences of 5‐nt and 12‐nt were used, and DNA sequences were encoded into term‐frequency vectors, position‐specific vectors, and motif‐based features for machine learning. In a recent study, a neural network‐based CNN model was used in the task of finding DNA‐SWCNT pairs that react specifically to serotonin, which using 18‐nt ssDNA.^[^ ^26^ ^]^ We used 30‐nt ssDNA, which is longer than previous studies, to understand the interaction of long‐length ssDNA with SWCNT. Also, we added 1D‐CNN, GRU, and transformer to the experiment, which are representative neural network models used in sequential data, to select models suitable for binding affinity prediction.

**Figure 4:**
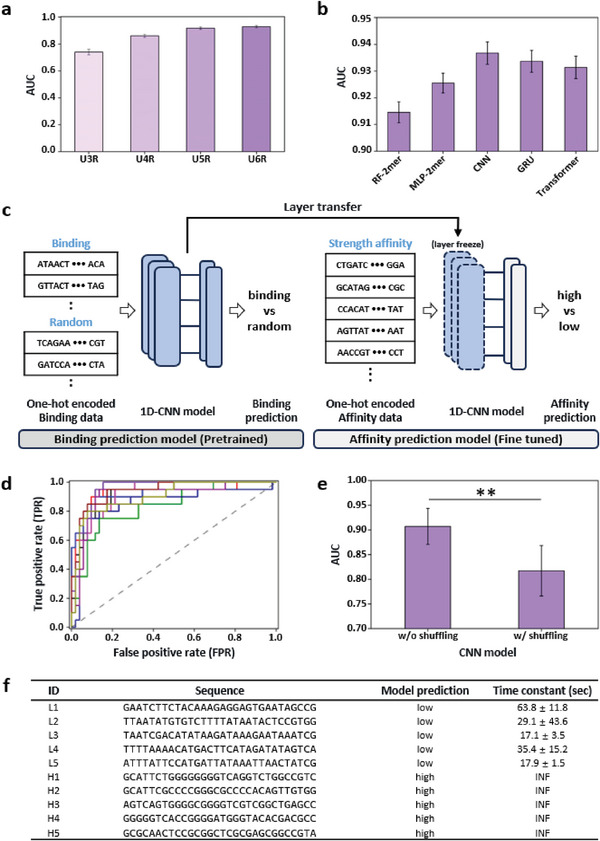
Prediction of the binding affinity between ssDNA and SWCNTs using machine‐learning models. a) Average prediction of the AUC for ssDNA binding of the five machine‐learning models from iteration rounds 3 to 6. b) Prediction AUCs of the five machine‐learning models at round 6. c) Overview of the proposed machine learning binding prediction and affinity prediction models. The affinity prediction model consists of layers transferred from the CNN model. d) ROC curve for the affinity prediction performance, with the 10 curves indicating 10 repeated experiments. Each ROC curve utilizes all prediction results from every fold, i.e., the probabilities for the affinity of all 72 sequences after a 4‐fold CV. e) Sequence shuffling experiment comparing the unshuffled and shuffled test sequences using the CNN model. **significant at *p* < 0.01. f) Validation results for the affinity prediction. Each column represents the test sequences, the strength prediction of our model, and the experimentally measured binding time constant. In ID, “L” denotes a low affinity sequence, and “H” denotes a high affinity sequence.

We leveraged machine‐learning techniques to predict and understand the dependence of SWCNT binding on the ssDNA sequences in a subset of the library data obtained in rounds 3, 4, 5, and 6. In each round, the top 10,000 sequences ranked by frequency were used as positive controls and 10,000 random sequences as negative controls. The random sequences are generated randomly by programming code and do not match any of the 10,000 binding positive sequences. The experiments were repeated 10 times with randomized splits for training, validation, and testing were set to 60%, 20%, and 20%, respectively. Binding prediction results showed the average of the 10 iterations in terms of accuracy, AUC, precision, recall, and F1 score at round 6 (Table S4, Supporting Information). The average performance of five machine‐learning models, random forest (RF),^[^ ^27^ ^]^ multi‐layer perceptron (MLP),^[^ ^28^ ^]^ convolutional neural network (CNN),^[^ ^29^ ^]^ gated recurrent unit (GRU),^[^ ^30^ ^]^ and Transformer,^[^ ^31^ ^]^ consistently increased as the rounds progressed (**Figure** **4**a). The prediction models in round 6 distinguished between binding sequences and random sequences with an AUC of approximately 92%. These results provide evidence that there are clear features of high‐affinity ssDNA sequences that machine learning can recognize, and ssDNA‐wrapped SWCNT constructs with these features survived more as the rounds progressed. In addition, we interpreted the 2‐mer random forest model in round 6 through Boruta‐SHAP.^[^ ^32^ ^]^ Dimers consisting of combinations of A and C and of T and G have high distribution changes as the round progresses and show high feature importance as well (Figures S2 and S6, Supporting Information). This supports that the changes in the distribution of nucleobases represent a feature of high‐affinity ssDNA.

To predict the binding affinity based on the time constant data presented in Table S2 (Supporting Information), we classified 72 sequences into high‐ and low‐affinity using a time constant threshold of 90 and used 20 (28%) high‐affinity sequences as positive examples and 52 (72%) low‐affinity sequences as negative examples (Table S5, Supporting Information). The time constant threshold of 90 s is chosen considering the discontinuous gap around 90 s in the time constant plot (Figure S7, Supporting Information) and to balance the number of high‐ and low‐affinity datasets as far as possible. We used transfer learning with our CNN model, which showed the best performance in the binding prediction experiments (Figure 4b,c). The experiments were repeated 10 times with 4‐fold cross‐validation, and the resulting prediction performances were plotted as ROC curves (Figure 4d). K‐fold cross‐validation partitioned the dataset into k subsets; for each iteration, one subset was chosen as the test set, while the remaining k‐1 subsets were used as the training set. This process was repeated *k* times to evaluate the model performance. The average performance over 10 repetitions had an AUC of 90.72% and an accuracy of 86.53%.

We conducted additional empirical validation to evaluate the binding strength prediction on ssDNA affinity. We predicted the affinity strength using our model for 100,000 random sequences that were not included in the training dataset. We then extracted five sequences each from the top and bottom of the probability values in our predictions and measured the time constant values for these sequences through actual surfactant displacement experiments. The experimental results showed successful predictions of binding affinity for all the test samples (Figure 4f). All sequences predicted as low strength exhibited time constants lower than the threshold value of 90 s. Similarly, all sequences predicted as high‐strength showed time constants recorded as “infinite (INF),” which were not measurable under 0.05 wt% SC conditions, indicating very strong affinity, with time constants higher than that of U6R‐40, the top sequence in round 6. These experimental results demonstrate the practicality and superiority of our model.

We also performed a sequence shuffling experiment for the nucleotide bases to determine the sequential dependence or correlation of the ssDNA sequences. The bases in the test sequence were randomly shuffled while maintaining the same base composition, and their performance was compared to that of the original sequence using the CNN model. The machine‐learning model showed significant performance degradation in experiments with shuffled sequences compared to those without shuffling (Figure 4e). These findings indicate that the order of the nucleotide bases is significant for the binding affinity of ssDNA sequences.

### 2.5 The Stability of ssDNA‐SWCNTs Against Nucleases Is Related to Their Binding Affinity

**Figure 5:**
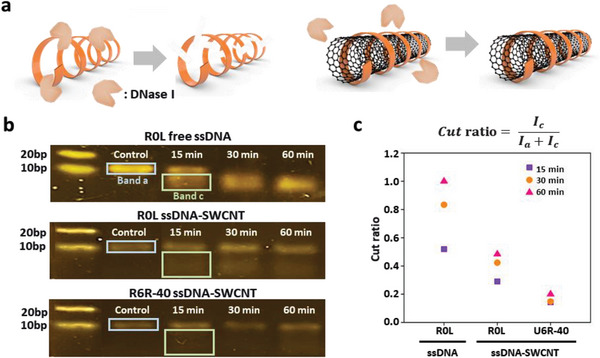
Highaffinity ssDNA‐SWCNT constructs are highly resistant to enzymatic DNA cleavage. a) Illustration of the DNase‐induced cleavage of free ssDNA and ssDNA on the SWCNT surface. b) DNase‐induced cleavage assessment of the free ssDNA (R0L mixture) and two ssDNA‐SWCNT (R0L, U6R‐40) after the incubation with DNase I as detected via 4% agarose gel electrophoresis. Band a and band c indicate the intact ssDNA and cleaved ssDNA fragments, respectively. c) Cut ratios indicate the proportion of cleaved ssDNA fragments over the total ssDNA.

For biological applications, oligonucleotides on ssDNA‐SWCNT constructs need to be resistant to nuclease digestion in cellular environments.^[^ ^33^ ^]^ Previous reports showed that ssDNAs on SWCNTs are more resistant to enzymatic cleavage than free ssDNAs (**Figure** **5**a).^[^ ^18^ ^]^ Nuclease resistance could be enhanced using high‐affinity ssDNA molecules, which hinder enzyme‐ligand interactions more effectively than low‐affinity ssDNA.^[^ ^33^, ^34^ ^]^

We compared the amount of ssDNA from low‐affinity (R0L mixture) and high‐affinity (U6R‐40) ssDNA‐SWCNTs enzymatically degraded by endonuclease DNase I. Two ssDNA‐SWCNT and free ssDNA samples were incubated with DNase I for 5, 15, 30, and 60 min following a previous protocol,^[^ ^18^ ^]^ and the ssDNA on SWCNTs were detached by heating at 95 °C. The degree of enzymatic cleavage was monitored via agarose gel electrophoresis (Figure 5b) and quantified using the cut ratio (CR) between the intact ssDNA (30‐nt) and shorter ssDNA fragments (<30‐nt) as shown in Equation 1 below:

(1) $$\mathrm{CR}\;=\;\frac{I_{c}}{I_{a}\;+\;I_{c}}$$

where *I_a_* is the fluorescence intensity of the intact ssDNA band and *I_c_* is the fluorescence intensity of the short‐cleaved ssDNA band. The relative CRs of U6R‐40‐SWCNT, R0L‐SWCNT, and free DNA at 60 min of incubation were 0.2, 0.48, and 1.00, respectively (Figure 5c). Oligonucleotides in high‐affinity ssDNA‐SWCNTs showed 5‐fold better resistance to DNase than free ssDNA and 2.5‐fold better resistance than low‐affinity ssDNA‐SWCNTs.

## 3 Discussion

In this study, we performed iterative screening to systematically select DNA molecules with a high binding affinity to SWCNT surfaces. The screening results expanded our fundamental knowledge of the interactions between DNA and SWCNTs to create stable DNA‐SWCNT constructs. Over six rounds of iteration, the proportion of A and C (31.6% and 33.5%, respectively) in the DNA library significantly increased compared to that of G and T (19.6% and 15.3%, respectively). This tendency is consistent with a previous report on the increased proportion of the AC motif over various selection rounds in an 18‐nt ssDNA selection protocol,^[^ ^18^ ^]^ indicating the positive influence of A and C on SWCNT binding affinity. Presumably, these sequence patterns related to binding affinity became more distinct, and it was inferred that the prediction performance through a machine‐learning model increased as the iteration rounds progressed.

Initially, we hypothesized that higher affinity DNA sequences would preferentially wrap SWCNT surfaces than lower affinity sequences during the formation of DNA‐SWCNT constructs. We verified this hypothesis using two DNA sequences, the high‐affinity Cy5‐modified (CG)_15_ sequence and the low‐affinity FAM‐modified U6R‐40 sequence. The fluorescent dyes Cy5 and FAM were applied to measure the concentrations of each DNA sequence. A 50:50 mixture of these two sequences was used to create the DNA‐SWCNT constructs. After sonication, the Cy5‐modified (CG)_15_ sequence attached to the SWCNTs at a larger proportion than the FAM‐modified U6R‐40 sequence, in the ratio 3:1 (Figure S8, Supporting Information). This result validates our initial hypothesis and underlies our systematic selection protocol for selecting high‐affinity DNA sequences from a random DNA library. It is noteworthy that some sequences with superior affinity than U6R‐40 (such as U6R‐40‐29T and (CG)_15_) were not included in the final round library. This discrepancy could be due to the following: 1) the limited amount of initial round 0 DNA library because we employed ≈6 × 10^16^ DNA sequences as the initial library, which might contain ≈10% (≈1.2×10^18^) total random unique 30‐nt DNA sequences, and (2) high‐affinity DNA sequences were not stripped off from the SWCNT surface during the detachment step, so those sequences were not observed in the subsequent libraries. It's possible to increase the detachment yield by using harsh conditions such as longer incubation time and the addition of competitive surfactants. That enhanced stripping method might be helpful in investigating the binding affinity of longer ssDNA in the future, which has a higher binding strength.

Previous studies have shown the sequence‐dependent interaction between DNA and SWCNTs, which can be utilized for SWCNT chiral sorting and biosensors.^[^ ^15^, ^35^ ^]^ In this study, we also observed that the binding affinity between DNA and SWCNTs was strongly related to the order of the bases in the DNA sequence. Scrambled U6R‐40‐S1, U6R‐40‐S2, and U6R‐40‐S3 have the same nucleobase composition as U6R‐40 but different nucleotide sequences. This finding is also consistent with the sequence shuffling experiment using machine learning. The shuffled test DNA sequences exhibited performance degradation compared to the original unshuffled test sequences, even though the sequences with and without shuffling had the same base composition. Therefore, the sequential patterns, i.e., specific order of bases, affect the binding affinity of DNA to SWCNTs.

Our results indicate that the binding affinity is influenced by both nucleotide sequence and base composition. Each DNA nucleobase (A, T, G, C) adsorbs on the SWCNT surface at different dissociation energies.^[^ ^8^, ^36^ ^]^ The dissociation enthalpies of specific sequences were estimated by simply summing the enthalpies of each nucleobase in the sequence (Figure S9, Supporting Information). A reasonably strong correlation (Pearson's coefficient = 0.6) was found between binding affinity and the estimated dissociation enthalpy, just as the time constant tended to increase as the enthalpy of the base increased. However, some results deviated from this trend, which were attributed to the base sequence alignment.

In the MD simulations, high‐affinity DNA sequences tend to form hydrogen bonds near the SWCNT surface. These high‐affinity DNA molecules also present more hydrogen bonds than low‐affinity DNA molecules. We observed many hydrogen bonds, such as 8A‐10T in U6R‐12 and 12C‐13G‐14C in U6R‐12. However, due to the limitation of coarse‐grained molecular dynamics simulation, these hydrogen bonds can't be categorized as either canonical or non‐canonical hydrogen bonds. Even though high‐affinity DNA sequences form hydrogen bonds, they tend to remain near the SWCNT surface, which might contribute to their restricted mobility. We quantitatively calculated the mobility of the DNA molecules by analyzing their mean square displacement (MSD) over time in the MD trajectories (Figure S10, Supporting Information). The MSD shows the deviation of the DNA position from a reference point as a function of time, as shown in equation 2 below:

(2) $$\mathrm{MSD}\;=\;\langle {\left|r\left(t\right)-r\left(0\right)\right|}^{2}\rangle \;=\frac{1}{N}\;\sum_{i\;=\;1}^{N}|r_{i}\left(t\right)-r_{i}\left(0\right){|}^{2}$$

where *N* is the target molecule number, ***r***_***i***_(*t*) is the center of mass position of the molecule at time t, and ***r**_i_* (0) represents all possible lag times. High‐affinity DNA sequences with high vdW energy (Figure S5, Supporting Information) have smaller MSD values than low‐affinity DNA sequences. Therefore, high‐affinity DNA exhibits lower mobility upon adsorption on the SWCNT surface than low‐affinity DNA molecules.

The formation of hydrogen bonding in the secondary structure near the SWCNT surface is crucial for stabilizing DNA‐SWCNT structures. Roxbury et al. utilized (6,5) chirality SWCNT in a water box containing sodium and phosphate ions, the DNA detached from the SWCNT surface, adopting a secondary wrapped configuration stabilized by hydrogen bonding between bases at distal ends of the DNA chain.^[^ ^9a^ ^]^ The hydrogen bonding close to the surface of the SWCNTs, whether through forming secondary structures or through stitching, is likely to enhance the stability of DNA on the SWCNT surface. This effect may decrease migration efficiency and further stabilize DNA adsorption.

Regarding the biological applications of ssDNA‐SWCNTs, it is known that ssDNA molecules wrapped around SWCNTs are more resistant to enzymatic degradation than free ssDNA.^[^ ^33^, ^37^ ^]^ Furthermore, we observed that high‐affinity DNA has better resistance against nuclease digestion and superior biostability in intracellular systems for drug delivery or biosensor probe applications. We conducted experiments to assess the biostability of DNA‐SWCNT constructs in a serum environment (Figure S11, Supporting Information). Notably, we observed significantly higher biostability in high‐affinity DNA‐SWCNT constructs compared to their low‐affinity counterparts. These results showed the critical role of affinity in influencing the biostability of DNA‐SWCNT constructs under physiological circumstances. This superior biostability enhances the potential utility of high affinity DNA‐SWCNT for applications requiring robust stability in serum or biological systems. We observed these desirable protective attributes (Figure 5c), which are important for the in vitro and in vivo application of DNA‐SWCNT constructs for monitoring intracellular biomolecules. It is feasible that high‐affinity DNA molecules on SWCNT surfaces could be applied for other ssDNA‐SWCNT technologies, such as stable anchor moieties for targeting and drug delivery and high‐yield chirality separation.

## 4 Conclusion

This study presents valuable insights into the intricate interactions between SWCNTs and ssDNA in terms of base composition and nucleotide sequence. The systematic selection approach demonstrated the robust binding affinity of 30‐nt ssDNA sequences to SWCNTs, significantly enhancing our comprehension of this interaction. MD simulations elucidated the critical roles of intramolecular hydrogen bonding and sequence patterns in determining binding strength. Moreover, our machine‐learning models provided predictive capabilities for binding affinity, supporting the design of tailored DNA‐SWCNT constructs. Our study not only makes a substantial contribution to our understanding of the interplay between ssDNA and SWCNTs but also offers practical avenues for harnessing these interactions in a wide range of advanced technologies. In the future, developing nanomaterials and devices with enhanced biostability will show promise in driving innovation in nanoelectronics and biotechnology.

## 5 Experimental Section

### Selection Protocol

The initial random ssDNA library had the general sequence 5′‐AGCGTCGAATACCACTAC‐N30‐GACCACGAGC TCCATTAG‐3′ (Integrated DNA Technologies) which consisted of 18‐nt primers for PCR amplification. For the first round, 10 µg of SWCNTs and 1 mg of the ssDNA library in PBS were bath‐sonicated for 2 min and then tip‐sonicated (Vibra cell Sonics CV18) for 30 min at 4‐W (50%) power in an ice bath. After sonication, the SWCNT dispersion was centrifuged for 60 min at 21,000 ×*g* to precipitate the undispersed SWCNTs. The supernatant containing the ssDNA‐SWCNT constructs was spin‐filtered using a 100‐kDa molecular weight cutoff centrifuge filter (Amicon ultra‐0.5, Millipore) at 6,000 rpm for 5 min with deoxyribonuclease (DNase)‐free water. This step was repeated five times to remove the unbound ssDNAs. To detach the bounded ssDNAs from the SWCNT surface, purified ssDNA‐SWCNTs were heated at 95 °C for 1 h in a dry bath. The ssDNA desorbed from the SWCNT surface was collected via centrifugation for 10 min at 21,000 ×*g* to precipitate the aggregated SWCNTs. The supernatant was amplified via PCR using a FAM‐modified forward primer (FAM‐AGCGTCGAATACCACTAC) and a biotinylated reverse primer (biotin‐CTAATGAGACTCGTGGTC) for the ssDNA library. Next, 5 U of Top DNA polymerase (Bioneer), 1× reaction buffer, 1 × 10^−6^ M forward primer, 1 × 10^−6^ M reverse primer, 1 × 10^−3^ M deoxynucleotide triphosphate (dNTPs), and the ssDNA library template (200 ng mL^−1^) were mixed in 10‐µL volumes in 96‐well reaction plates. A negative control without an ssDNA library template was also prepared. PCR amplification was conducted with the following cycling conditions: initial denaturation for 60 s at 94 °C, N cycles of denaturation for 20 s at 94 °C, annealing for 30 s at 50 °C, and extension for 45 s at 72 °C, and final extension step at 72 °C for 300 s. The number of cycles, N, was determined from a preparative PCR run, which yielded maximal ssDNA product and negligible PCR byproduct (usually between 10 and 20 cycles). Next, 100 µL of the PCR product was purified with a GeneJET PCR Purification kit (Thermo Fisher Scientific) to prepare sequencing libraries. The PCR products were confirmed via electrophoresis in a 4% agarose gel (Bioneer) stained with SYBR Gold in 1× tris‐borate‐EDTA buffer (run for 20 min at 110 V). After electrophoresis, the DNA bands were observed under a blue LED light. To separate ssDNA from the PCR product, 2 mL of streptavidin‐coated beads (Pierce High‐Capacity Streptavidin Agarose, Thermo Scientific) were placed in a sintered glass Buchner funnel (pore size <10 µm) and washed with 10 mL of DNase‐free water. The PCR product was incubated with the beads for 30 min to bind dsDNA, and the beads were washed twice with 20 mL of water. To elute the FAM‐labeled ssDNAs, 8 mL of a 0.2 M NaOH solution was incubated for 10 min and filtered. The eluted ssDNAs were desalted with a NAP‐10 desalting column (Glen Research) and concentrated using a freeze‐dryer. The amount of ssDNA was quantified by measuring their absorbance at 260 nm. Finally, 2–3 nmol of ssDNA was obtained and used for the next round.

### DNA Sequencing and Analysis

Sequencing libraries obtained via PCR were prepared by using TruSeq Nano DNA kit and sequenced in a paired‐end 151 bp approach with Illumina NovaSeq 6000 platform at ROKIT Genomics. The libraries generated 5‐6 million raw sequencing data from rounds 3 to 6. The sequences were preprocessed to filter out the unique random 30‐nt regions fixed by two primers and determined the sequence counts. All experiments were performed using ssDNA sequences without PCR primer regions. For the k‐mer analysis, the frequencies of the monomer, dimer, and trimer sequences in the top 20,000 sequences of each round were calculated and presented using an RF model.

### Optical Characterization of SWCNTs

For all spectroscopy studies, a 721 nm laser (PSU‐H‐LED laser power supply) was used as the excitation light source, and detection was performed using an InGaAs photodiode array detector (Ocean Insight, NIRQuest). The absorption spectra were measured with a UV–VIS–NIR absorption spectrophotometer (Labotech, V‐1600).

### Fabrication of ssDNA‐SWCNT Constructs

Here, 1 mg of HiPCo SWCNTs and 100 nmol of 1 × 10^−3^ M ssDNA were mixed in 0.9 mL of PBS. The resulting mixture was bath‐sonicated for 2 min and tip‐sonicated (SONICS VCX‐130) for 30 min at 4 W power in an ice bath. After sonication, the ssDNA‐SWCNT solution was centrifuged for 1 hour at 21,000 ×*g* to precipitate the undispersed SWCNTs, and the supernatant containing the solubilized ssDNA‐SWCNTs was collected. The supernatant was spin‐filtered with 100‐kDa MWCO centrifuge filters at 6,000 rpm for 5 min with DNase‐free water. Spin filtration was performed thrice to remove unbound ssDNA. The ssDNA‐SWCNT constructs were collected, and diluted with PBS, and the concentration of the ssDNA‐SWCNT solution was calculated by measuring its absorbance at 632 nm using the SWCNT extinction coefficient (0.036 mg L ^−1^ cm^−1^).

### Evaluation of the Spectral shift of ssDNA‐SWCNTs Induced by SC Surfactant Replacement

SC‐induced solvatochromic shift assays via ligand exchange were performed to quantify the binding affinity of ssDNA to SWCNTs. For fluorescence monitoring, 5 mg L^−1^ ssDNA‐SWCNT samples were prepared in DI water and loaded onto a NIR fluorescence spectrometer. The time‐resolved fluorescence spectra were obtained before and after adding 4 or 10 µL of 5 wt% SC dissolved in DI water. The spectra were measured every 20 s for a total of ≈10 min with 0.02 wt% or 0.05 wt% (final concentration) of SC added to each sample after 100 s. The spectral blue shift was measured for the (9,4) chirality fluorescence peak and the time‐dependent spectral shift at approximately 1129 nm was quantified.

### DNase Resistance Assay

For the endonuclease resistance assay, 8 mg L^−1^ of ssDNA‐SWCNTs was incubated with 1 U of DNase I (Thermo Scientific, RNase‐free) in MgCl_2_ buffer (1×) at 37 °C (total volume, 100 µL). Both ssDNA‐SWNT constructs (N30, U6R‐40) and free DNA (U6R‐40) were treated with DNase I, and aliquots were collected at the 5‐, 15‐, 30‐, and 60‐min time points. Aliquots were added to a 50 × 10^−3^ M EDTA solution and heated at 95 °C for 10 min before separating on a 6% agarose gel. The CR was calculated using ImageJ software to quantify DNA cleavage.

### Hydrogen Bond Disruption Using Urea

To monitor hydrogen bond disruption in the DNA molecules, 5 mg L^−1^ ssDNA‐SWCNT constructs (C30, U6R‐12, U6R‐40) were prepared at 21 °C with or without 3 M urea. The time‐resolved fluorescence spectra were measured before and after adding 10 µL of 5 wt% SC for 10 min. The spectral blueshift was measured for the (9,4) chirality fluorescence peak, and the time‐dependent spectral shift at approximately 1129 nm was quantified.

### The MARTINI Force Field

In this work, CGMD simulations were conducted using GROMACS version 2020.3,^[^ ^23^ ^]^ with the MARTINI force field 2 employed as the coarse‐grained model. The MARTINI force field is widely recognized and utilized in molecular simulations due to its unique approach of representing groups of atoms as a single bead. The MARTINI force field employs 4‐to‐1, 3‐to‐1, and 2‐to‐1 mapping, corresponding to grouping four, three, and two heavy atoms into a single bead, respectively. This reduction in computation enables faster simulations compared to typical all‐atom force field‐based MD simulations. Additionally, the integration time step is more than 10 times larger than an all‐atom force field, allowing the simulation of larger systems at a higher speed.^[^ ^24^ ^]^ The armchair carbon nanotube (CNT) model employed in this study was parameterized with a diameter of approximately 1.2 nm.^[^ ^38^ ^]^ The ssDNA molecules were parameterized using MARTINI version 2.1_dna.^[^ ^39^ ^]^ In the case of water, P4 particles were mixed with 10% BP4 beads, which acted as antifreeze beads.^[^ ^24^ ^]^ To represent the strong interaction between CNT and ssDNA, the C1 bead was used for CNT.^[^ ^40^ ^]^

### Molecular Dynamics Simulations

In the MD simulations, CNT was positioned at the center of the xy plane within a simulation box with dimensions of 20 × 20 × 20.759 nm. To generate infinitely long nanotubes along the z‐axis, the two ends of the CNT were connected with a bond, and a position restraint was applied to the z‐axis to prevent its movement. Then, the system was simulated using the NPxyT ensemble (semi‐isobaric ensemble).^[^ ^41^ ^]^ Four ssDNA molecules were selected for this simulation: two with a high time constant (U6R‐40 and U6R‐12) and two with a low time constant (U6R‐06 and U6R‐18). Each ssDNA molecule consists of a 30‐nt sequence (right‐handed). Each simulation cell consists of a single CNT (416 beads), a single ssDNA molecule (190‐194 beads depending on the type of ssDNA), 61,739 P4 beads (water), and 6,859 BP4 beads (antifreeze beads), amounting to approximately 70,000 system beads. Following the system setup, the energy of the system was minimized through consecutive energy minimization steps using the steepest descent and conjugated gradient integrators. The total energy minimization value was set to under 500 kJ mol^−1^. After energy minimization, a 20‐ns NPxyT simulation was performed using the v‐rescale thermostat^[^ ^42^ ^]^ for temperature coupling and the Berendsen barostat^[^ ^43^ ^]^ for pressure coupling. During this step, the simulation was conducted at a temperature of 300 K and a pressure of 1 bar. This step facilitated the stabilization of pressure and box size fluctuations. To achieve the system's equilibrium state, the Parrinello‐Rahman barostat^[^ ^44^ ^]^ was performed for pressure coupling after NPxyT simulation for 500 ns, keeping the temperature and pressure constant. The dt was set to 0.01 ps for all NPxyT simulations. After 300 ns of simulation time, the system was considered to have reached the equilibrium state, so the last 200 ns of the simulation trajectory was used for the analysis. In the simulations, a short‐range cutoff of 1.1 nm was applied to both vdW and Coulombic interactions. Long‐range interactions were calculated using the reaction field algorithm.^[^ ^45^ ^]^ The number of hydrogen bonds in DNA bases was evaluated based on the distance between DNA base pair beads. If the distance between DNA base pair beads was 3.5 Å or less, it was considered to form a hydrogen bond. The classification of “near H‐bond” or “far H‐bond” was analyzed over the last 200 ns (2,000 frames) of the simulation and the results were expressed as percentages.

### Datasets and Preprocessing Methods

10,000 binding sequences were used from the ssDNA library and 10,000 random sequences for the binding prediction experiments. Binding sequences can be sufficiently obtained through the iteration experiments, but we did not perform measurements of binding strength. To measure the binding affinity strength, experimental separation needs to be conducted for each ssDNA sequence‐SWCNT complex, leading to a scarcity of samples. For affinity strength experiments, we prepared 72 sequences for modeling and 10 sequences for further empirical validation. DNA sequences were preprocessed into two types: k‐mer‐based and one‐hot encoding‐based. For the traditional machine‐learning and MLP models, the k‐mer‐based features composed of the frequencies of the k‐length subsequences were used. The value of *k* varies from 1‐mers to 5‐mers, having the 4^k^ feature dimension. In the case of 1‐mers, only the composition frequency was considered, not the base order. Increasing the value of *k* has the advantage of handling longer subsequences but has the disadvantage of higher dimensionality and increased sparsity of the feature vectors. We experimented with k‐mers from 1‐ to 5‐ and observed that 2‐mer showed the best performance in RF and MLP (Table S6, Supporting Information). Therefore, we used the 2‐mer as a reference for subsequent comparative analyses. For neural network models that can consider sequential dependency, we performed one‐hot encoding for the A, C, G, and T bases of the 30‐nt sequences. Each base position was represented as a binary vector of size 4 (representing A, C, G, and T), and only the corresponding base has a value of 1, while the others have a value of 0. Each one‐hot encoded 30‐bp‐long DNA sequence has a dimension of 30×4, preserving the order of the nucleotide bases. Detailed data preprocessing, experimental setups, and model parameters for machine learning are described in Note S1 (Supporting Information).

### Machine‐Learning Models

Five machine‐learning models were used for the binding prediction experiments: the traditional machine‐learning model random forest (RF) and four artificial neural network (ANN)‐based models: multi‐layer perceptron (MLP), gated recurrent unit (GRU), convolutional neural network (CNN), and transformer. RF and MLP use the k‐mer‐based features as input, while the remaining ANN‐based models use the one‐hot‐encoded data as input. Due to the limited number of samples for strength prediction and the traits of the prediction tasks, we expanded our binding prediction model to affinity strength prediction by employing a transfer learning scheme: binding prediction as pre‐training and affinity strength prediction as fine‐tuning. These two tasks have some similarities and differences. Binding sequences can have different levels of strength, so sequences with very strong affinity may not be found in the ssDNA library, presumably due to their inherent self‐coiling or other reasons. The CNN, which exhibited the best performance in the binding prediction, was employed as the basis for the affinity prediction. The CNN architecture consists of five 1D‐conv layers and a fully connected layer, where the first three conv layers were frozen, and the remaining layers were fine‐tuned for the transferred task. The affinity prediction model is publicly available at the following link: http://service.k‐medai.com/ssdna4cnt.

## Conflict of Interest

The authors declare no conflict of interest.

## Author Contributions

D.L., J.L., and W.K. contributed equally to this work. S.J., S.K., Y.K., and S.K. conceived the idea and supervised the overall project. D.L. and Y.S. synthesized the construct and performed selection experiments. D.L. experimentally screened the binding affinity of ssDNA‐SWCNT constructs and analyzed the overall data. W.K. and Y.K. performed and analyzed the MD simulation. J.L., J.P., and S.K. performed and analyzed the ML models. All authors contributed to the discussion of results and writing the manuscript.

## Supporting information

Supporting Information

Supplemental Movie 1: MD simulation of SWCNT with U6R‐40 ssDNA

Supplemental Movie 2: MD simulation of SWCNT with U6R‐12 ssDNA

Supplemental Movie 3: MD simulation of SWCNT with U6R‐06 ssDNA

Supplemental Movie 4: MD simulation of SWCNT with U6R‐18 ssDNA

## Data Availability

The data that support the findings of this study are available from the corresponding author upon reasonable request.

## References

[1] a) M. Zhao , Y. Chen , K. Wang , Z. Zhang , J. K. Streit , J. A. Fagan , J. Tang , M. Zheng , C. Yang , Z. Zhu , W. Sun , Science 2020, 368, 878; 32439791 10.1126/science.aaz7435

[2] a) M. Kim , C. Chen , P. Wang , J. J. Mulvey , Y. Yang , C. Wun , M. Antman‐Passig , H. B. Luo , S. Cho , K. Long‐Roche , L. V. Ramanathan , A. Jagota , M. Zheng , Y. Wang , D. A. Heller , Nat. Biomed. Eng. 2022, 6, 267; 35301449 10.1038/s41551-022-00860-y PMC9108893

[3] X. M. Tu , S. Manohar , A. Jagota , M. Zheng , Nature 2009, 460, 250. 19587767 10.1038/nature08116

[4] a) T. Jiang , C. A. Amadei , N. Gou , Y. Lin , J. Lan , C. D. Vecitis , A. Z. Gu , Environ. Sci.: Nano 2020, 7, 1348; 33537148 10.1039/d0en00230e PMC7853656

[5] a) J. Budhathoki‐Uprety , R. E. Langenbacher , P. V. Jena , D. Roxbury , D. A. Heller , ACS Nano 2017, 11, 3875; 28398031 10.1021/acsnano.7b00176 PMC5511501

[6] a) J. Ackermann , J. T. Metternich , S. Herbertz , S. Kruss , Angew. Chem., Int. Ed. 2022, 61, e202112372; 10.1002/anie.202112372 PMC9313876 34978752

[7] D. Roxbury , A. Jagota , J. Mittal , J. Phys. Chem. B 2013, 117, 118. 23199189 10.1021/jp309523a

[8] a) F. Albertorio , M. E. Hughes , J. A. Golovchenko , D. Branton , Nanotechnology 2009, 20, 395101; 19724110 10.1088/0957-4484/20/39/395101 PMC2739102

[9] a) D. Roxbury , A. Jagota , J. Mittal , J. Am. Chem. Soc. 2011, 133, 13545; 21797248 10.1021/ja204413v

[10] R. R. Johnson , A. T. Johnson , M. L. Klein , Nano Lett. 2008, 8, 69. 18069867 10.1021/nl071909j

[11] M. Zheng , A. Jagota , E. D. Semke , B. A. Diner , R. S. McLean , S. R. Lustig , R. E. Richardson , N. G. Tassi , Nat. Mater. 2003, 2, 338. 12692536 10.1038/nmat877

[12] R. R. Johnson , A. Kohlmeyer , A. T. Johnson , M. L. Klein , Nano Lett. 2009, 9, 537. 19161335 10.1021/nl802645d

[13] R. R. Johnson , A. T. Johnson , M. L. Klein , Small 2010, 6, 31. 19943252 10.1002/smll.200901481

[14] a) Y. Yang , M. Zheng , A. Jagota , npj Comput. Mater. 2019, 5, 3;

[15] X. Gong , N. Renegar , R. Levi , M. S. Strano , npj Comput. Mater. 2022, 8, 1.

[16] a) S. M. Copp , P. Bogdanov , M. Debord , A. Singh , E. Gwinn , Adv. Mater. 2014, 26, 5839; 25043854 10.1002/adma.201401402

[17] D. Roxbury , J. Mittal , A. Jagota , Nano Lett. 2012, 12, 1464. 22375694 10.1021/nl204182b

[18] S. Jeong , D. Yang , A. G. Beyene , J. T. Del Bonis‐O'Donnell , A. M. M. Gest , N. Navarro , X. Sun , M. P. Landry , Sci. Adv. 2019, 5, eaay3771. 31897432 10.1126/sciadv.aay3771 PMC6920020

[19] F. F. Bergler , S. Stahl , A. Goy , F. Schoppler , T. Hertel , Langmuir 2016, 32, 9598. 27575847 10.1021/acs.langmuir.6b02759

[20] J. Q. Zhang , M. P. Landry , P. W. Barone , J. H. Kim , S. C. Lin , Z. W. Ulissi , D. H. Lin , B. Mu , A. A. Boghossian , A. J. Hilmer , A. Rwei , A. C. Hinckley , S. Kruss , M. A. Shandell , N. Nair , S. Blake , F. Sen , S. Sen , R. G. Croy , D. Y. Li , K. Yum , J. H. Ahn , H. Jin , D. A. Heller , J. M. Essigmann , D. Blankschtein , M. S. Strano , Nat. Nanotechnol. 2013, 8, 959. 24270641 10.1038/nnano.2013.236 PMC5051352

[21] a) H. T. Maune , S. P. Han , R. D. Barish , M. Bockrath , W. A. Goddard, III , P. W. Rothemund , E. Winfree , Nat. Nanotechnol. 2010, 5, 61; 19898497 10.1038/nnano.2009.311

[22] G. Suresh , S. Padhi , I. Patil , U. D. Priyakumar , Biochemistry 2016, 55, 5653. 27657980 10.1021/acs.biochem.6b00309

[23] a) S. Pronk , S. Pall , R. Schulz , P. Larsson , P. Bjelkmar , R. Apostolov , M. R. Shirts , J. C. Smith , P. M. Kasson , D. van der Spoel , B. Hess , E. Lindahl , Bioinformatics 2013, 29, 845; 23407358 10.1093/bioinformatics/btt055 PMC3605599

[24] S. J. Marrink , H. J. Risselada , S. Yefimov , D. P. Tieleman , A. H. de Vries , J. Phys. Chem. B 2007, 111, 7812. 17569554 10.1021/jp071097f

[25] Z. Lin , Y. Yang , A. Jagota , M. Zheng , ACS Nano 2022, 16, 4705. 35213805 10.1021/acsnano.1c11448

[26] a) P. Kelich , S. Jeong , N. Navarro , J. Adams , X. Q. Sun , H. H. Zhao , M. P. Landry , L. Vukovic , ACS Nano 2022, 16, 736; 34928575 10.1021/acsnano.1c08271

[27] L. Breiman , Mach. Learn. 2001, 45, 5.

[28] D. E. Rumelhart , G. E. Hinton , R. J. Williams , Nature 1986, 323, 533.

[29] A. Krizhevsky , I. Sutskever , G. E. Hinton , Commun. ACM. 2017, 60, 84.

[30] K. Cho , B. V. Merrienboer , C. Gulcehre , D. Bahdanau , F. Bougares , H. Schwenk , Y. Bengio , Proceedings of the 2014 Conference on Empirical Methods in Natural Language Processing 2014,1724.

[31] A. Vaswani , N. Shazeer , N. Parmar , J. Uszkoreit , L. Jones , A. N. Gomez , L. Kaiser , I. Polosukhin , Proceedings of the 31st International Conference on Neural Information Processing Systems, 2017, 6000.

[32] M. B. Kursa , W. R. Rudnicki , J. Stat. Software 2010, 36, 1.

[33] Y. Wu , J. A. Phillips , H. Liu , R. Yang , W. Tan , ACS Nano 2008, 2, 2023. 19206447 10.1021/nn800325a PMC2658617

[34] a) N. K. Mehra , V. Mishra , N. K. Jain , Biomaterials 2014, 35, 1267; 24210872 10.1016/j.biomaterials.2013.10.032

[35] a) F. Yang , M. Wang , D. Q. Zhang , J. Yang , M. Zheng , Y. Li , Chem. Rev. 2020, 120, 2693; 32039585 10.1021/acs.chemrev.9b00835

[36] F. K. Brunecker , F. Schoppler , T. Hertel , J. Phys. Chem. C 2016, 120, 10094.

[37] S. J. Wu , N. Schuergers , K. H. Lin , A. J. Gillen , C. Corminboeuf , A. A. Boghossian , ACS Appl. Mater. Interfaces 2018, 10, 37386. 30277379 10.1021/acsami.8b12287

[38] M. Vogele , J. Kofinger , G. Hummer , Faraday Discuss. 2018, 209, 341. 29974904 10.1039/c8fd00011e

[39] J. J. Uusitalo , H. I. Ingolfsson , P. Akhshi , D. P. Tieleman , S. J. Marrink , J. Chem. Theory Comput. 2015, 11, 3932. 26574472 10.1021/acs.jctc.5b00286

[40] J. Li , H. R. Zhang , M. F. Yu , Q. Li , T. Y. Zhang , Z. C. Xue , H. Sun , J. Electroanal. Chem. 2022, 917, 116380.

[41] S. C. Lin , J. Q. Zhang , M. S. Strano , D. Blankschtein , Soft Matter 2014, 10, 5991. 24992310 10.1039/c4sm00974f

[42] G. Bussi , D. Donadio , M. Parrinello , J. Chem. Phys. 2007, 126, 014101. 17212484 10.1063/1.2408420

[43] H. J. C. Berendsen , J. P. M. Postma , W. F. Vangunsteren , A. Dinola , J. R. Haak , J. Chem. Phys. 1984, 81, 3684.

[44] a) M. Parrinello , A. Rahman , J. Appl. Phys. 1981, 52, 7182;

[45] L. Onsager , J. Am. Chem. Soc. 1936, 58, 1486.

