# Supplementary material for: Systematic Selection of High‐Affinity ssDNA Sequences to Carbon Nanotubes
Source: Adv Sci (Weinh). 2024 Jun 25;11(32):2308915. doi: 10.1002/advs.202308915 (PMC11348070; doi:10.1002/advs.202308915)
Supplement: Supplementary file 1 — Supporting Information [file ADVS-11-2308915-s004.docx]

**Supplementary Information**

**Systematic Evolution of High Affinity ssDNA Sequences**

**to Carbon Nanotubes**

Dakyeon Lee, Jaekang Lee, Woojin Kim, Yeongjoo Suh, Jiwoo Park, Sungjee Kim*, YongJoo Kim*, Sunyoung Kwon*, Sanghwa Jeong*

**Note S1.** Detailed experimental setups for machine learning.

(Data preparation)

For the binding prediction, we prepared 10,000 binding sequences and 10,000 random sequences for each round. Binding sequences were from high-through sequencing of ssDNA library through evolutionary experiments and random sequences were randomly generated using programming code. The dataset was split into training, validation, and test with a ratio of 60:20:20 and the experiments were repeated 10 times.

For the affinity prediction, we prepared 72 sequences and quantitively measured their binding affinity through displacement experiments. Due to the limited number of samples, the experiment was conducted using 4-fold cross-validation, and the cross-validation was repeated 10 times.

(Libraries for machine learning)

We used the TensorFlow library (version 2.9.1) and Keras library (version 2.9.0) packages for neural network-based models: MLP, GRU, CNN, and Transformer, and the Scikit-learn library (version 1.0.2) package for conventional machine learning model: RF.

(Model architectures and parameters)

For the binding prediction, we tested five models: RF and MLP using k-mer-based features, and GRU, CNN, and Transformer using the one-hot encoded features. RF and MLP were tested using k-mers ranging from 1-mer to 5-mer.

- RF was experimented with default parameters.

- MLP with 2-dense layers (100, 30 dims), CNN with 5-Conv1D layers (128, 64, 32, 16, 8 dims) and flattened, GRU with 3-bidirectional GRU layers (30, 20, 10 dims), flattened, and 1-dense layer (80 dim), and Transformer with positional encoding block, 3-transformer block (all 4 dims) and 3-dense layers (128, 64, 32 dimes) and flattened were constituted, and all models had output layer as 1-dense layer (2-dim) for classification. Dropouts were placed between layers. We used the following detailed settings: the activation function, ReLU for all hidden layers, and softmax for the last output layer; Adam optimizer; loss function as binary cross-entropy.

For the affinity prediction, we used the same CNN architecture which showed the best performance in the binding prediction; CNN with 5-Conv1D layers (128, 64, 32, 16, 8 dims) and flattened, and output layer as 1-dense layer (2-dim) for classification. The weight parameters of the first 3-Conv1D layers from the binding prediction were transferred to the affinity prediction and frozen during training.


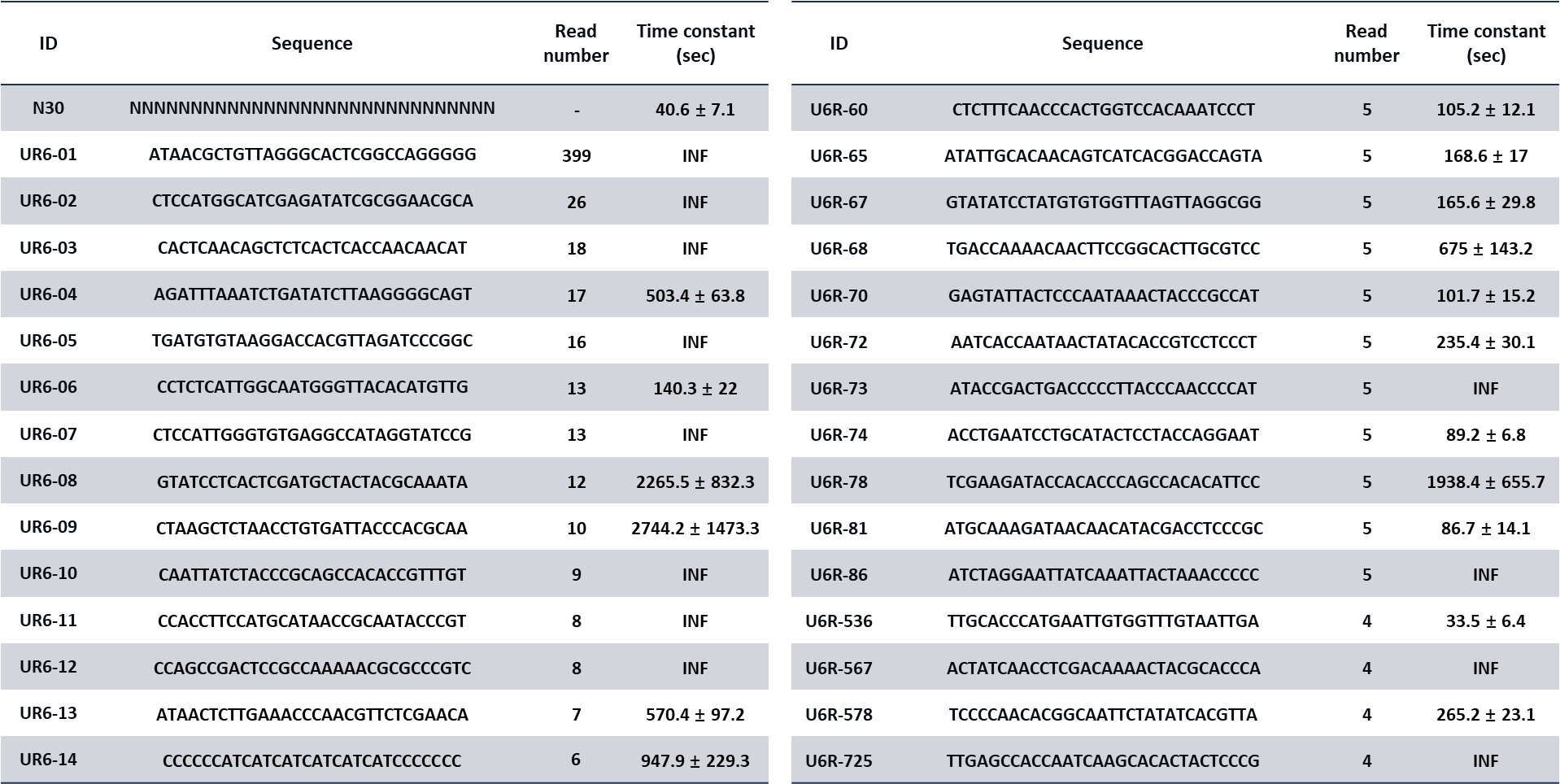


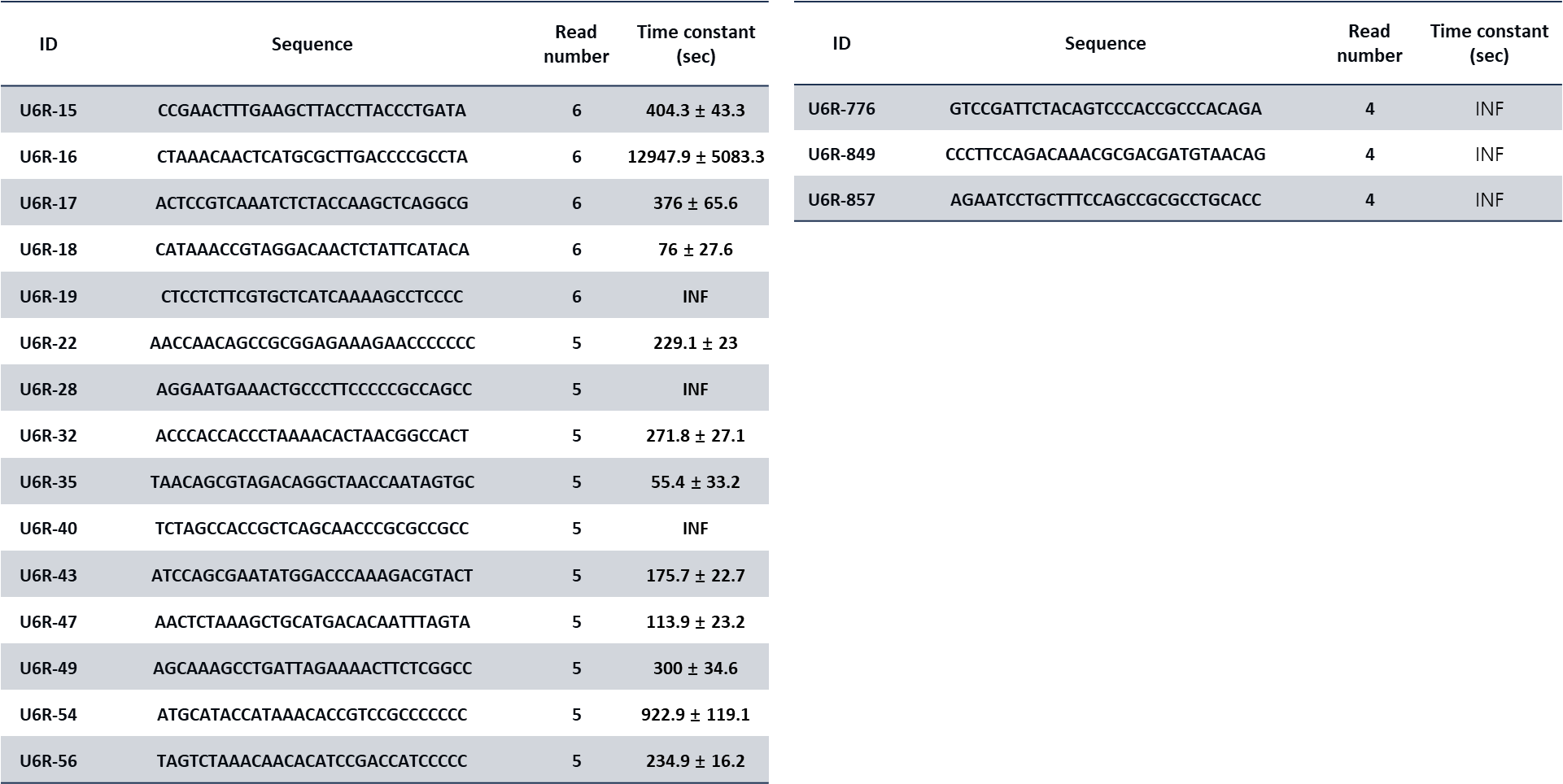


**Table S1.** The 47 sequences shown were tested to measure the displacement time constant for the binding affinity of the round 6 library at 0.02 wt% SC conditions. INF indicates a non-solvatochromic shift; this reflects high binding affinity on the SWCNT surface at 0.02 wt% SC condition, making it difficult to calculate the time constant.


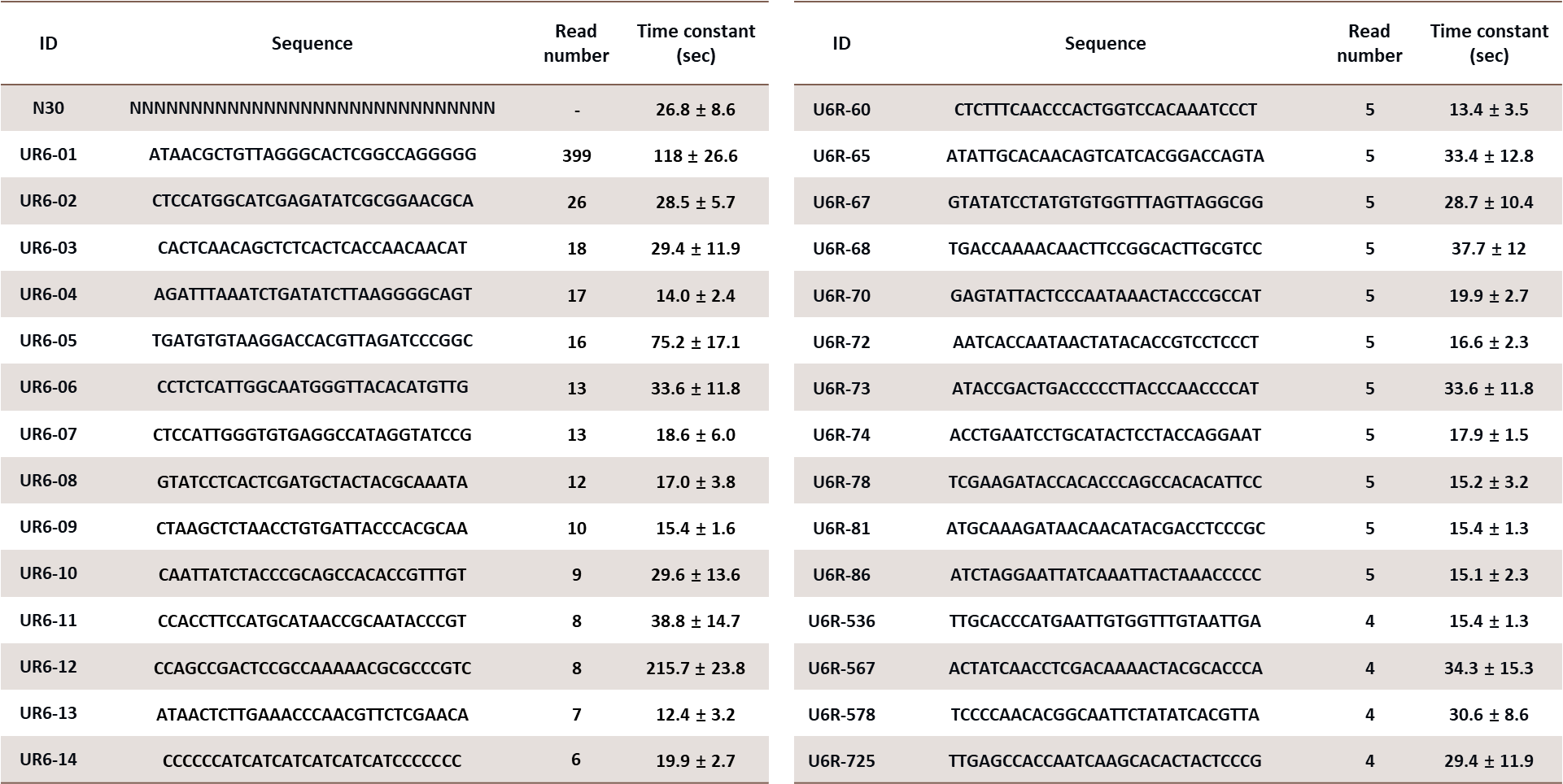


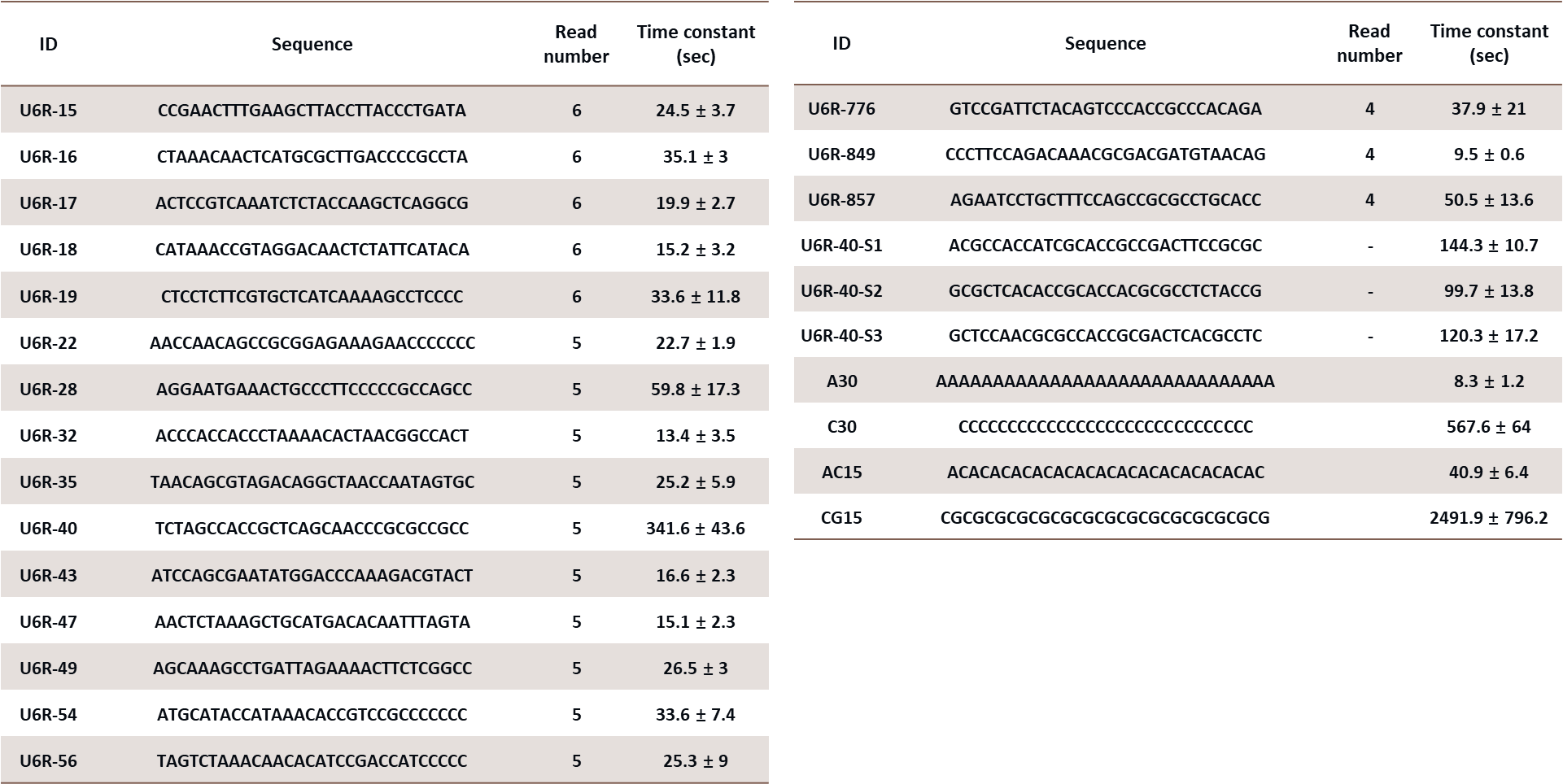


**Table S2.** The 47 sequences shown were tested to measure the displacement time constant for the binding affinity of the round 6 library at 0.05 wt% SC conditions.


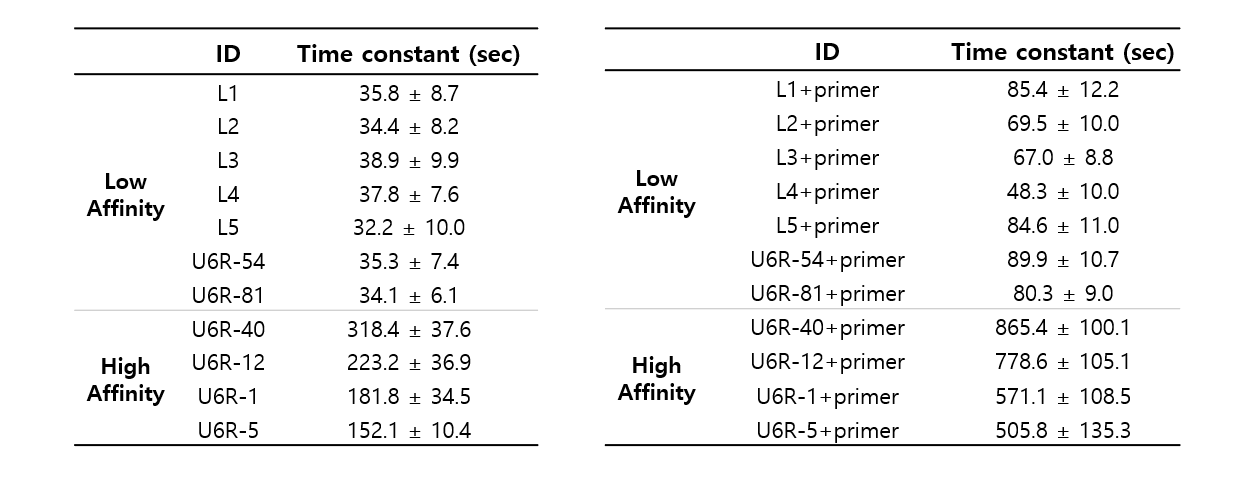


**Table S3.** The binding affinity in the presence of primer under SC 0.05 wt%. The high affinity sequences U6R-40, U6R-12, U6R-5, and U6R-1 showed an increase in time constant. The low affinity sequences L1, L2, L3, L4, L5, U6R-54, and U6R-81 maintain the low time constant in the presence of primer. Overall, high and low affinity sequences increased by a time constant of 2-5 folds in the presence of primers.


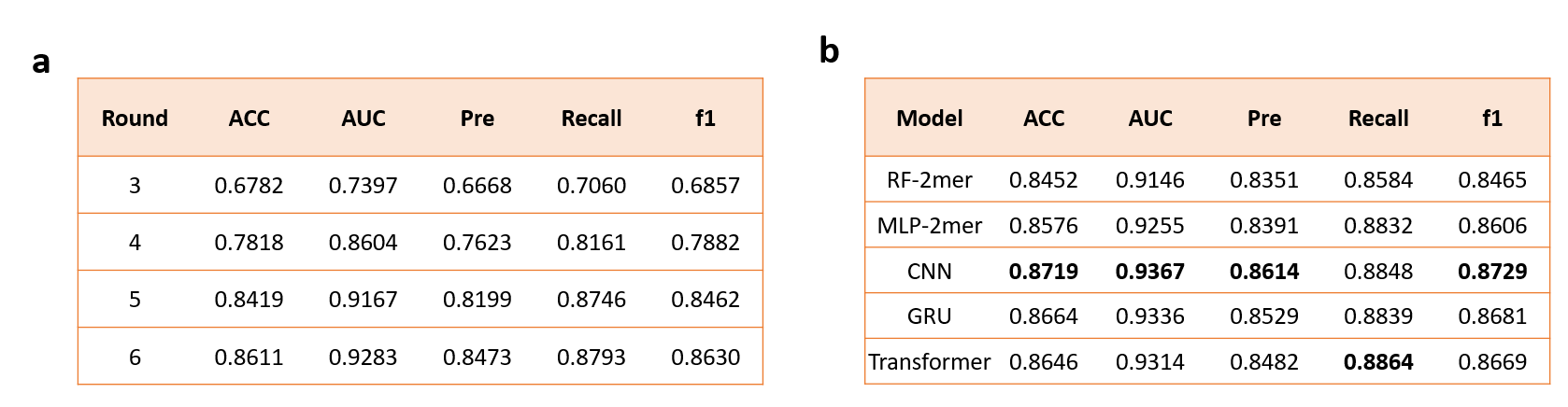


**Table S4. a)** Averages of the five models from the 10 repetitions in terms of accuracy, AUC, precision, recall, and F1 score at rounds 3, 4, 5, and 6. **b)** Prediction performance for ssDNA binding using the five machine-learning models at round 6. The results were evaluated for 10 repetitions. Values in bold indicate the best performance in each model.


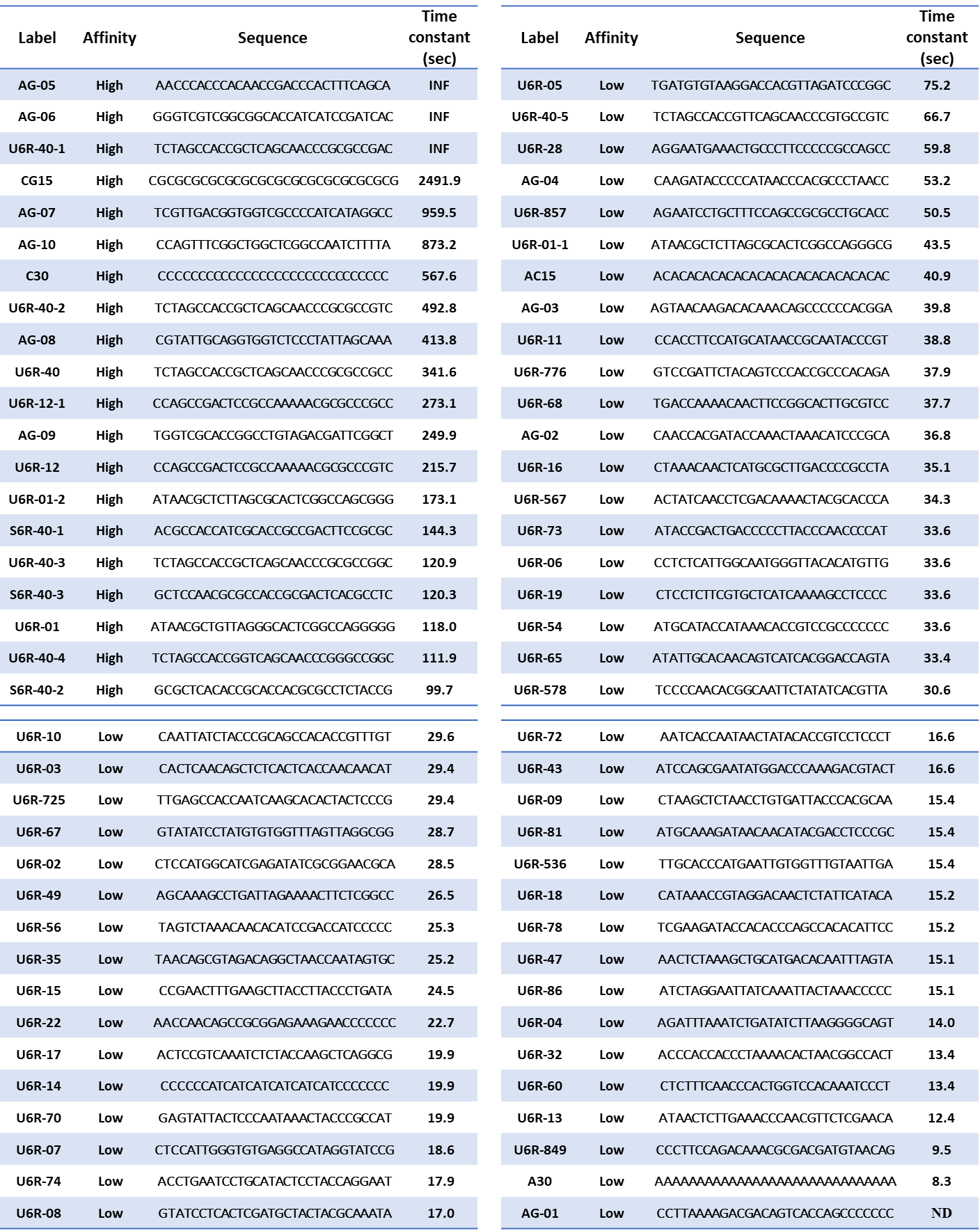


**Table S5.** Affinity labels, sequences, displacement time constant values for affinity prediction in 72 sequences. Based on the threshold 90, 20 high-affinity and 52 low-affinity sequences were prepared for affinity classification. The 10 artificially generated sequences were also used and denoted as AG-X, where X stands for number. The value that was not detected is noted as 'ND’.


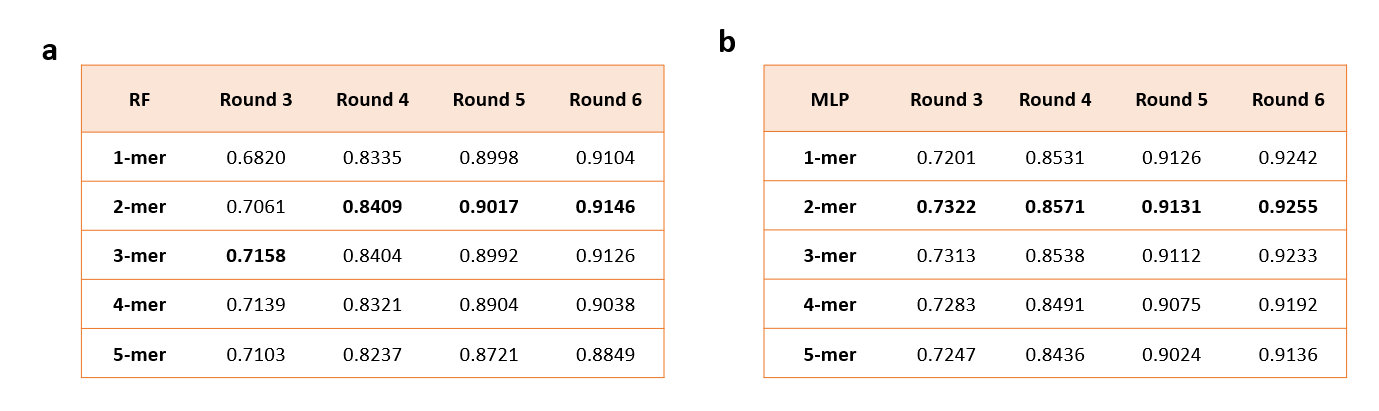


**Table S6.** Experimental results of k-mer based binding prediction in terms of AUC. The k-mers ranged from 1-mer to 5-mer, with conducted in rounds 3 to 6. Values in bold indicate the best performance among k-mers in each round. **a)** results using random forest (RF). **b)** results using multi-layer perceptron (MLP).


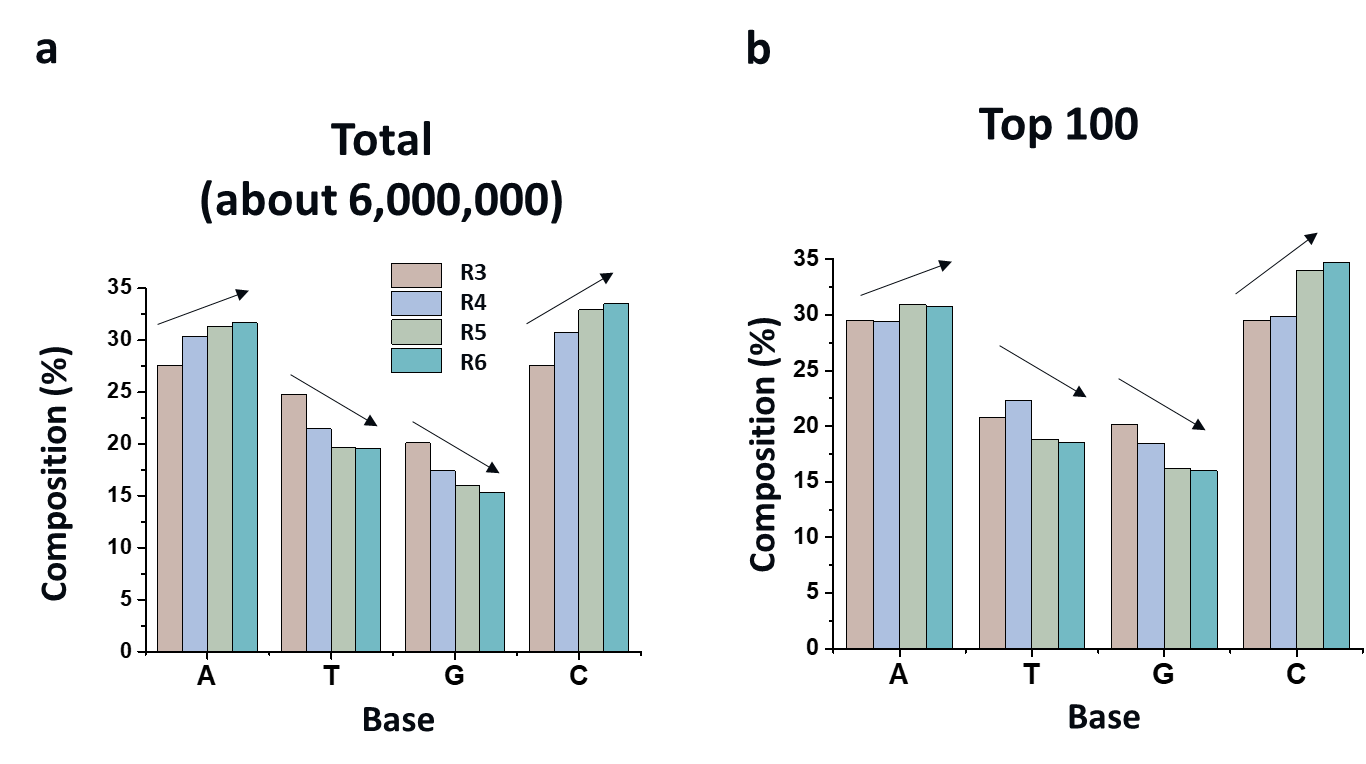


**Figure S1.** Distribution of ATGC composition. **a)** ATGC composition of each library across various iteration rounds and **b)** ATGC distribution for the top 100 ranked sequences in each library.


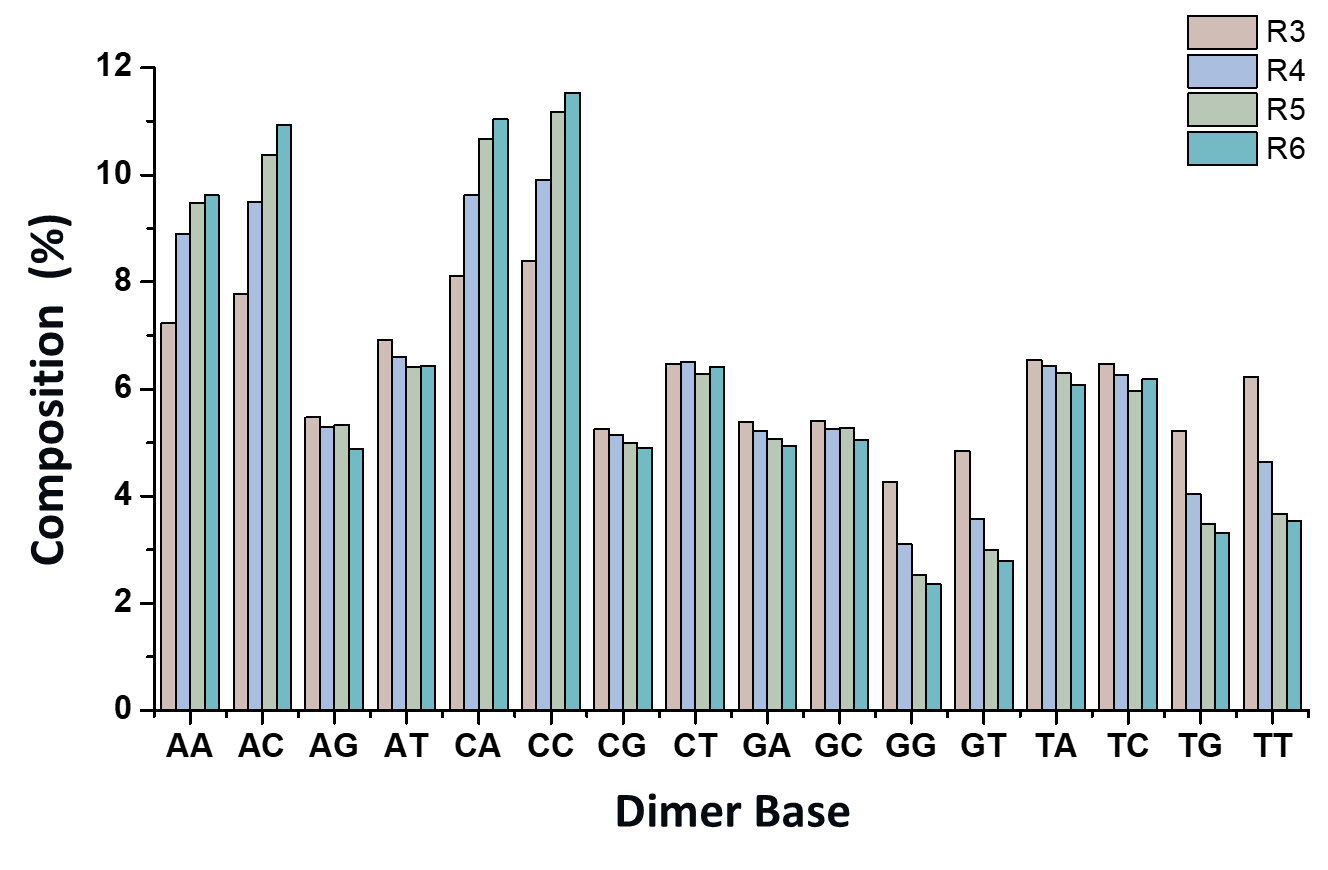
**Figure S2.** Distribution of dimer sequences (all 16 combinations) in the top 20,000 sequences of each library.

In this study, we quantitatively evaluated the degree of solvatochromic shift on the kinetics of ssDNA desorption after surfactant treatment. The solvatochromic shift is ssDNA sequence-dependent. The time constant of the solvatochromic shift reflects the time for ssDNA to be replaced by SC on the single-walled carbon nanotube (SWCNT) surface. We assumed the dynamic follows a first-order model and exponential decay from which the time constant ($\tau$) was determined.

$$\boldsymbol{ssDNA}\boldsymbol{-}\boldsymbol{SWCNT}\boldsymbol{+}\boldsymbol{nSC} \boldsymbol{\to}\boldsymbol{SC}\boldsymbol{-}\boldsymbol{SWCNT}\boldsymbol{+}\boldsymbol{mDNA}$$

$$\boldsymbol{rate}\boldsymbol{= -}\frac{\boldsymbol{d}\left[ \boldsymbol{ssDNA}\boldsymbol{-}\boldsymbol{SWCNT} \right]}{\boldsymbol{dt}}\boldsymbol{=-}\frac{\boldsymbol{\Delta}\boldsymbol{\lambda}}{\boldsymbol{dt}}$$

$$\boldsymbol{\Delta}\boldsymbol{\lambda}\boldsymbol{=}{\boldsymbol{\Delta}\boldsymbol{\lambda}}_{\boldsymbol{max}}\boldsymbol{\times(}\boldsymbol{e}^{\boldsymbol{-}\frac{\boldsymbol{\Delta}\boldsymbol{t}}{\boldsymbol{\tau}}}\boldsymbol{-}\boldsymbol{1}\boldsymbol{)}$$

**Figure S3**. To determine the degree of SC-induced ssDNA desorption, we used the reaction equations shown, assuming a first-order model, to quantitatively analyze the binding affinity of ssDNAs to SWCNTs. The ﬂuorescence corresponding to the (9,4)-chiral SWCNTs was examined for SC-induced blue-shift at 1,133 nm, which can be used to quantify the strength of the interaction between ssDNA and SWNTs. After adding SC, the (9,4) peak shifted to 1115 nm, which is the (9,4) peak of the SC-dispersed SWCNT, over time (~ 800 s).


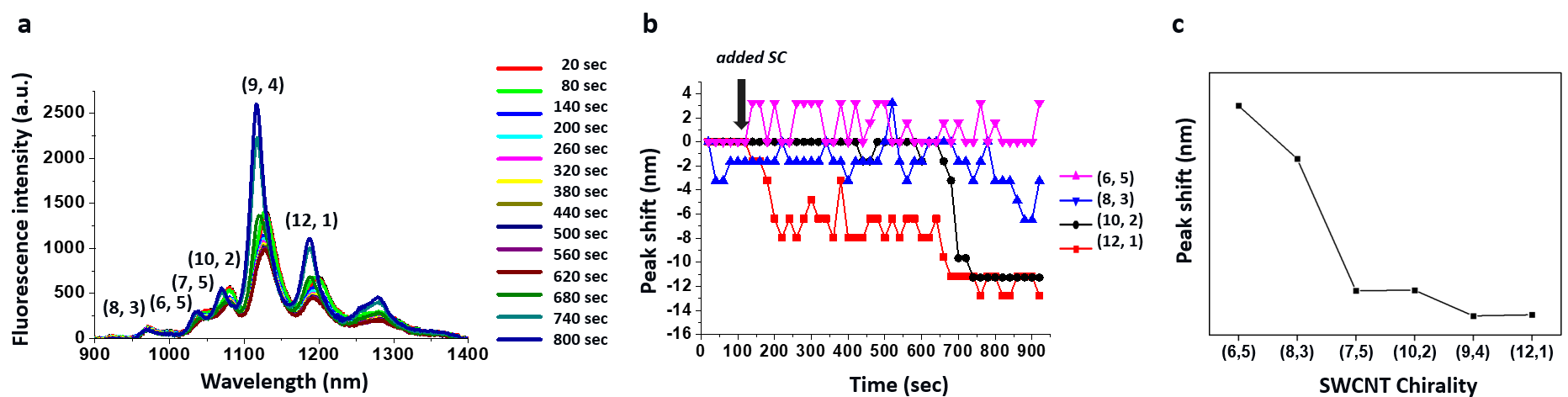


**Figure S4. a)** Time-dependent relative changes in ssDNA-SWCNT(U6R-13) ﬂuorescence after incubation with 0.02 wt % sodium cholate. **b)** Emission peak shift for each SWCNT chirality shows diameter dependence. c) The change in each fluorescence peak for various SWCNT chirality was measured at initial wavelengths: 972.45 nm of (6,5), 980.55 nm of (8,3), 1046.76 nm of (7,5), 1080.55 nm of (10,2), 1128.68 nm of (9,4), and 1198.99 nm of (12,1)). The shifts ranged from 0 nm for the smallest diameter, (6,5), to -12.82 nm for the largest diameter, (12,1).


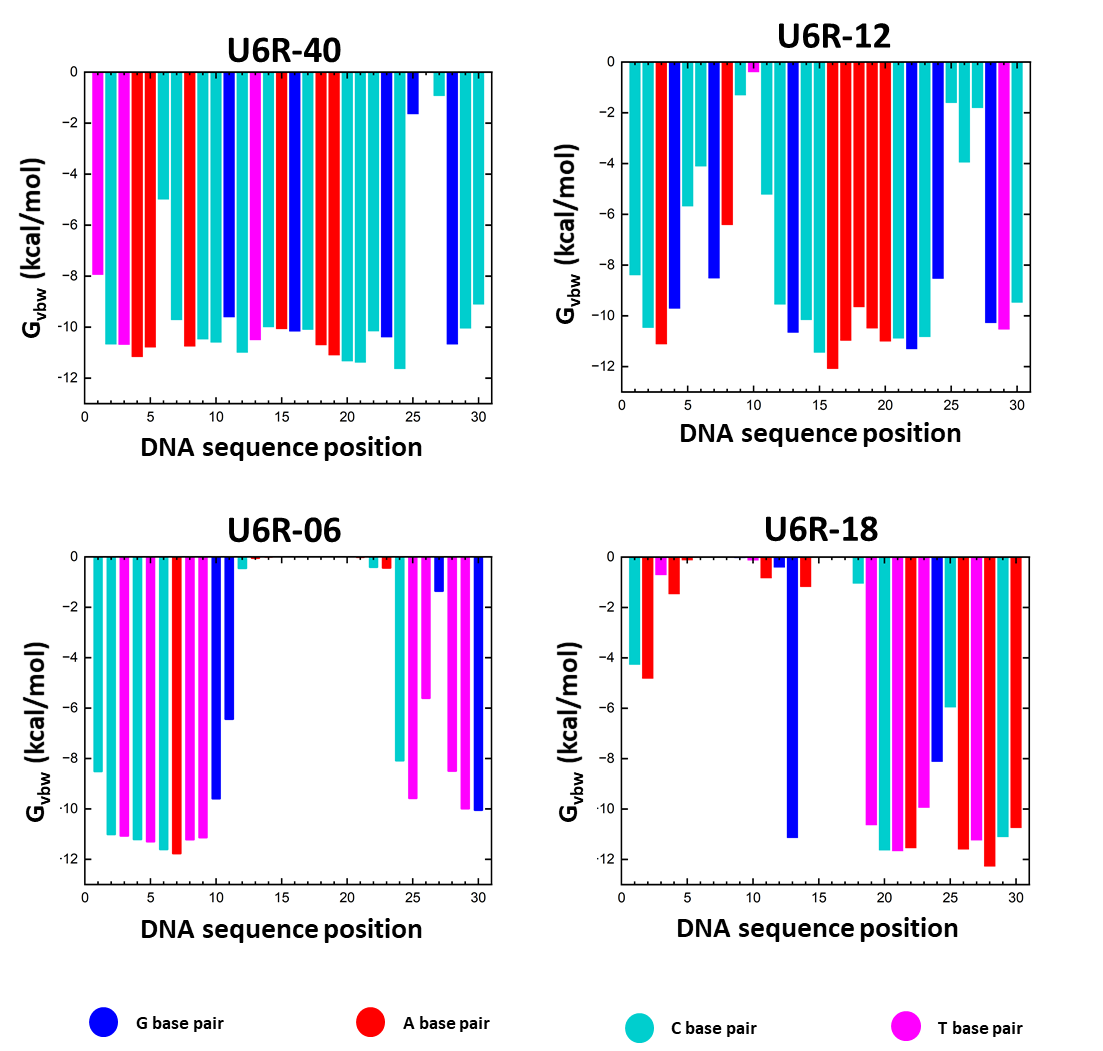


**Figure S5.** The van der Waals (vdW) energies of each nucleobase in the U6R-40, U6R-12, U6R-06, and U6R-18 sequences after reaching equilibrium. Negative vdW energies show positive binding affinity to SWCNT.


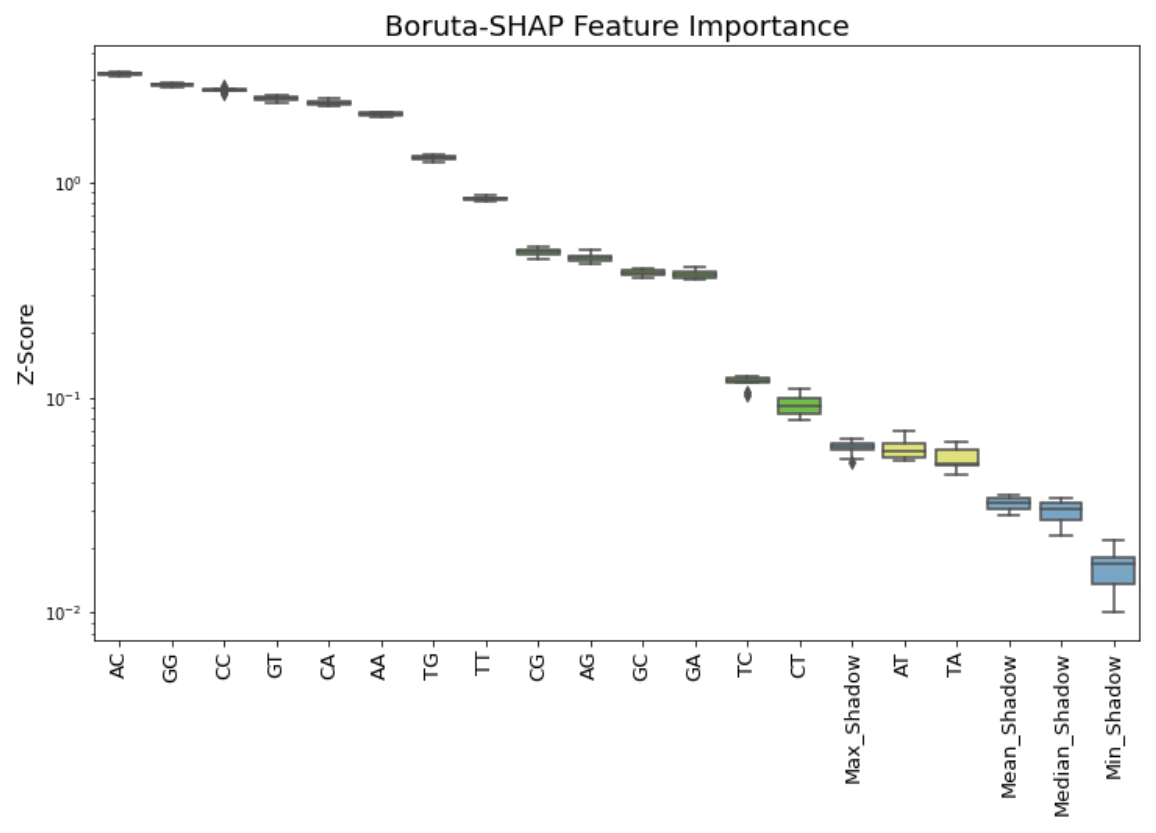


**Figure S6.**Feature importance of the 2-mer Random Forest calculated via Boruta-SHAP at Round 6 with 10 repetitions. The shadow means a pseudo feature randomly permuted of feature values, and the importance of meaningful features is shown to be higher than the importance of shadow features.


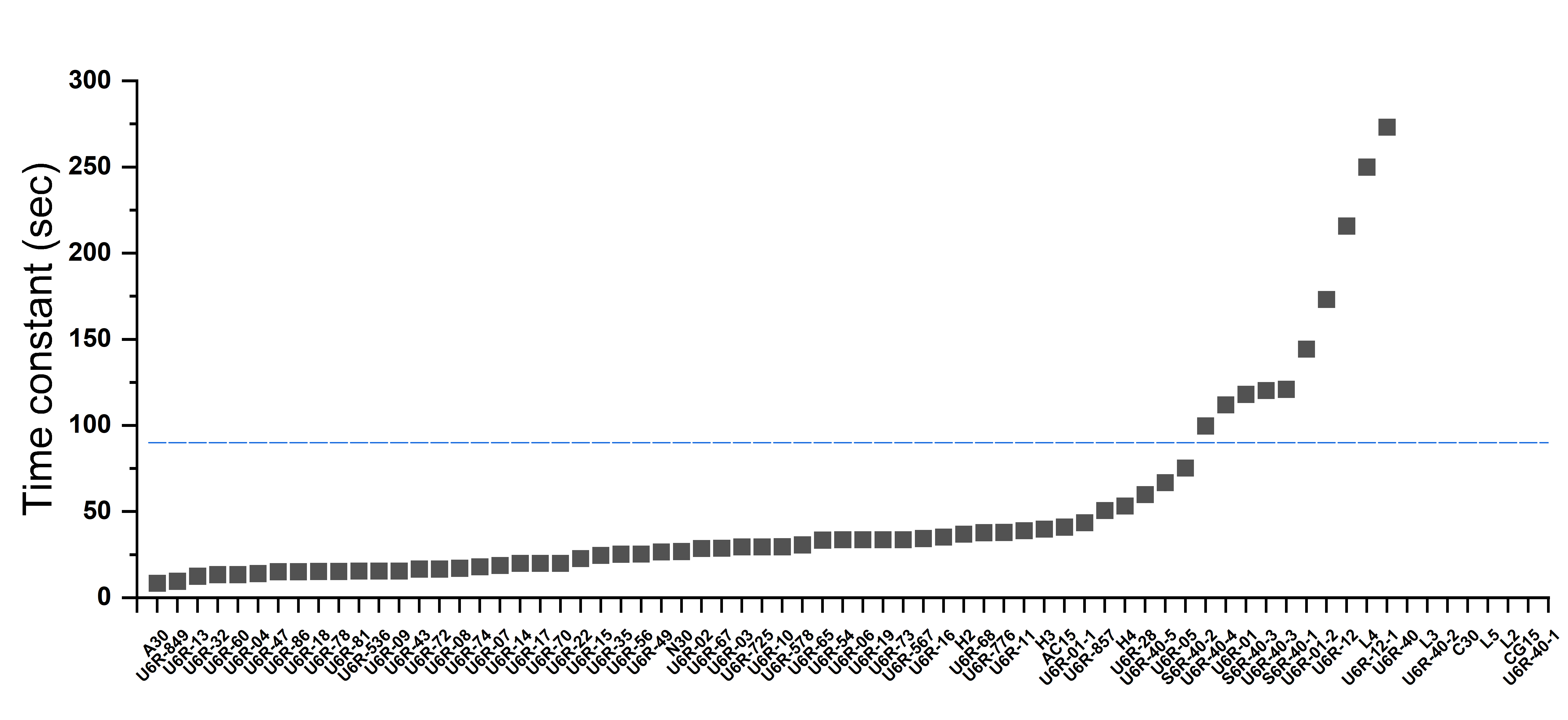


**Figure S7.** The time constants of sequences in the training data set were plotted with alignment of ascending order. The threshold of 90 in machine learning model was determined based on the observation of gap around 90s in the data around that point in time.


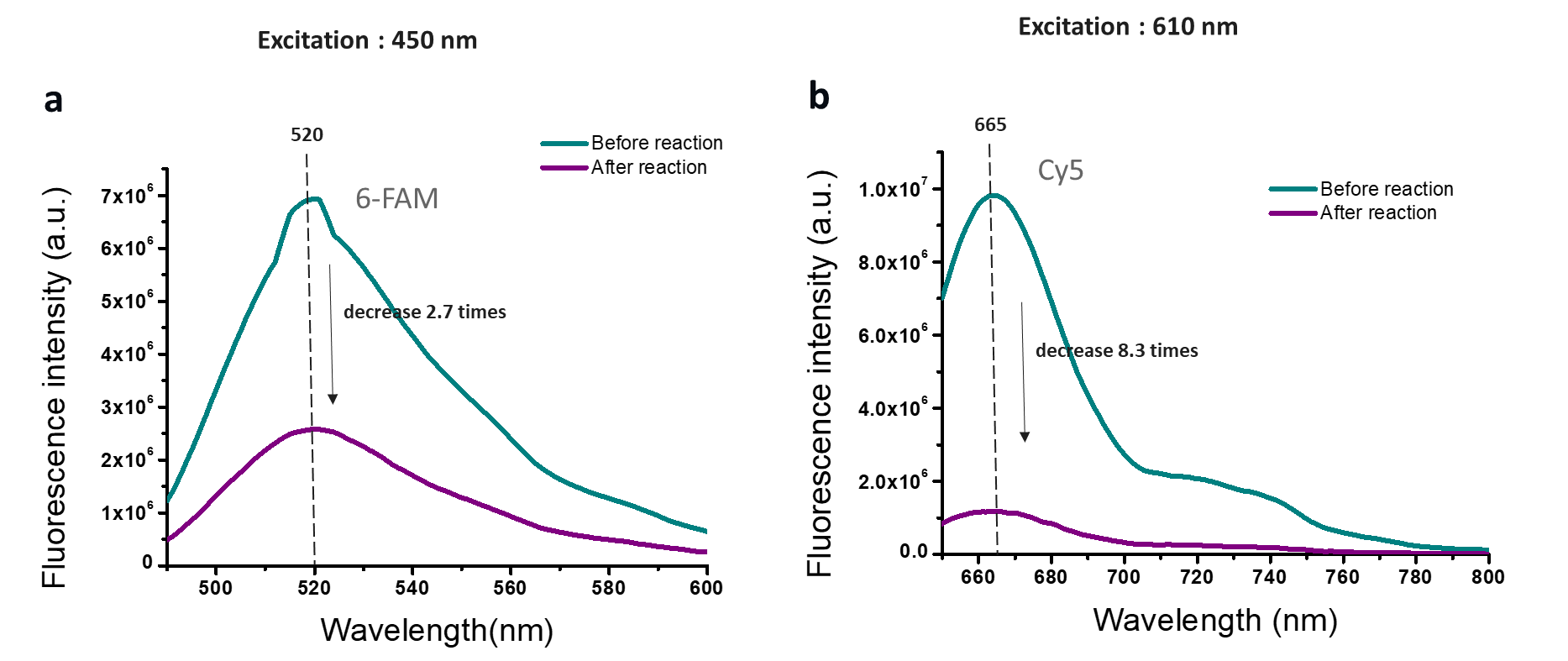


**Figure S8.** Test for competition between 5′ Cy5-(CG)_15_ DNA and 5′ 6-FAM-U6R-40 DNA on the SWCNT surface. The fluorescence intensity of the Cy5 and 6-FAM dyes decreased at excitation wavelengths of **a)** 450 nm and **b)** 610 nm after reaction.


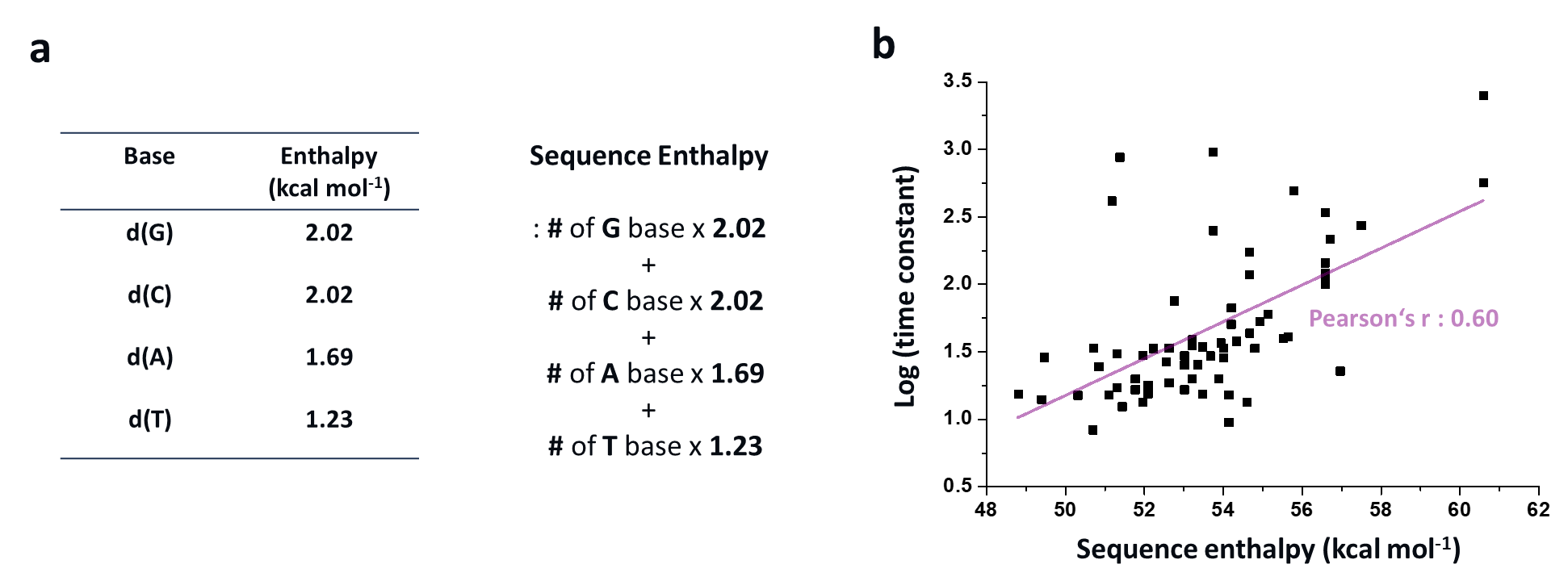


**Figure S9. a)** The enthalpies of the nucleotide bases were determined to calculate the total enthalpy of the DNA sequence. **b)** Correlation between the total enthalpy and time constant of DNA sequences.


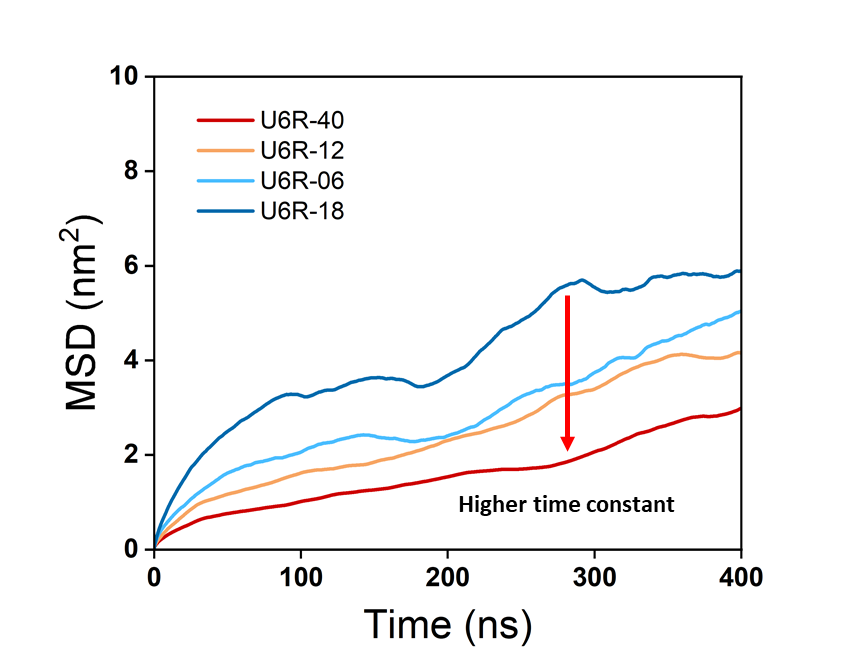


**Figure S10.** MSD plot of the U6R-40, U6R-12, U6R-06, and U6R-18 sequences as simulation time progress. High-affinity DNA sequences demonstrate a tendency to experience more restricted movement on the CNT surface compared to their low-affinity counterparts.


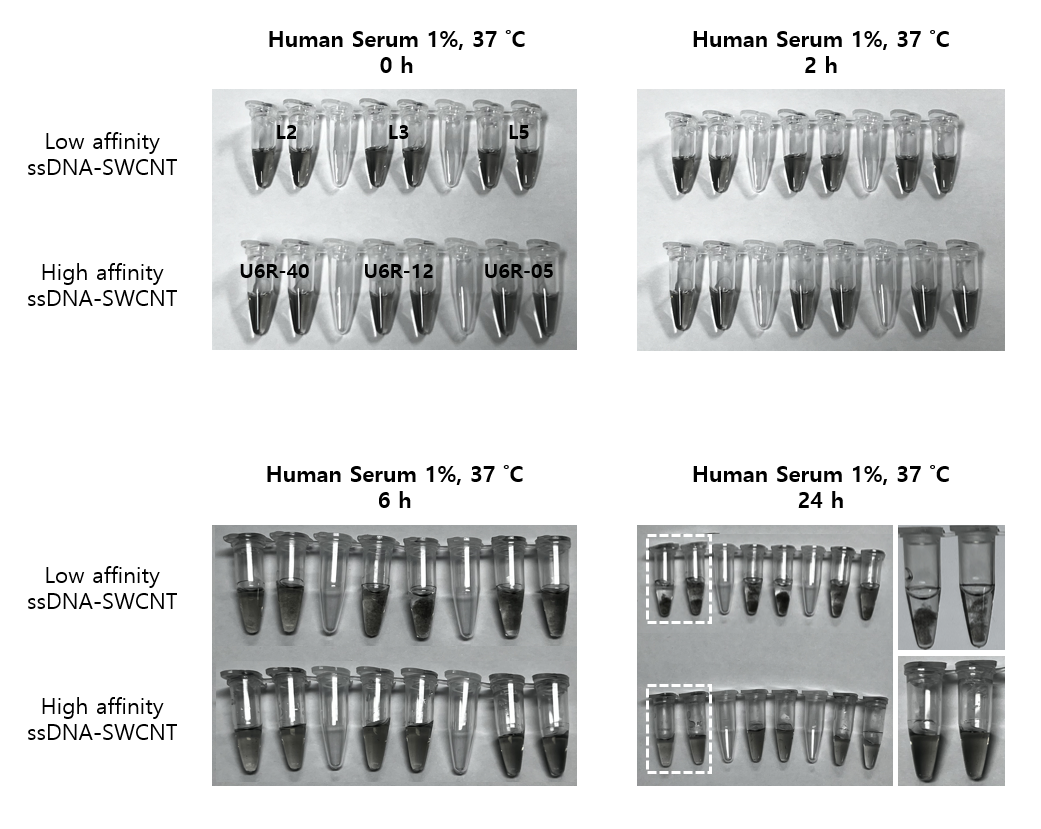


**Figure S11.** The biostability of DNA-SWCNT constructs in a serum environment. The low-affinity sequences (L2, L3, and L5) and high-affinity sequences (U6R-40, U6R-12, and U6R-5) were incubated in 1% human serum at 37℃. After 6 hours, the low-affinity DNA-SWCNT constructs exhibited aggregation, indicating reduced stability in the serum environment. In contrast, the high-affinity DNA constructs demonstrated biostability, and maintain a high level of dispersion.
